# DNA methylation signatures of cervical pre‐invasive and invasive disease: An epigenome‐wide association study

**DOI:** 10.1002/ijc.35406

**Published:** 2025-03-10

**Authors:** Sarah J. Bowden, Barbara Bodinier, Maria Paraskevaidi, Ilkka Kalliala, Maria Nasioutziki, Laura Burney Ellis, Ruben Colindres Zuehlke, James M. Flanagan, Maria Kyrgiou, Marc Chadeau‐Hyam

**Affiliations:** ^1^ Department of Metabolism, Digestion and Reproduction IRDB, Faculty of Medicine, Imperial College London London UK; ^2^ Department of Surgery & Cancer IRDB, Faculty of Medicine, Imperial College London London UK; ^3^ West London Gynaecology Cancer Centre, Hammersmith Hospital, Imperial Healthcare NHS Trust London UK; ^4^ Department of Epidemiology and Biostatistics, School of Public Health, Imperial College London London UK; ^5^ Department of Obstetrics & Gynaecology University of Helsinki, and Helsinki University Hospital Helsinki Finland; ^6^ Molecular Cytopathology Laboratory 2nd Obstetrics & Gynecology Clinic Hippokration General Hospital, Aristotle University of Thessaloniki Thessaloniki Greece

**Keywords:** cervical cancer, cervical intraepithelial neoplasia, epigenome, EWAS, human papillomavirus, methylation, PAX

## Abstract

Epigenetic alterations are essential in the development of cancers, while epigenome‐wide exploration in cervical cancer has been limited. In this epigenome‐wide association study (EWAS) we explore differential DNA methylation signatures associated with CIN (cervical intraepithelial neoplasia) grade 3 and cervical cancer to better understand potential drivers and biomarkers of cervical carcinogenesis. 247 women were recruited between 2014 and 2020 (*N* = 119 benign, *N* = 74 CIN3/CGIN [cervical glandular intraepithelial neoplasia] and *N* = 54 cancer). Methylation signatures were obtained from exfoliated cervical cells and sequenced using the Illumina 850 k array. Logistic regression and conditional analyses were used to test for independent associations between Cytosine‐phosphate‐Guanine (CpG) sites and case–control status, with adjustment for batch, chip, age, and human papillomavirus (HPV) status. 409 CpG sites were strongly associated with CIN3/cancer (*p*‐value <5 × 10^−8^). Following conditional analysis, two CpG sites located in *PAX1* (cg16767801) and *NREP‐AS1* genes (cg23642047) were independently associated with case status, yielding an area under the curve (AUC) of 0.92 (AUC = 0.97 for invasive disease). In a validation dataset (CIN3 only) *PAX1/NREP‐AS1* yielded a combined AUC of 0.77. Methylation markers offer promise for use in cervical screening, particularly as triage tests and self‐sampling. We have identified a novel combined methylation marker that offers a high accuracy for the detection of CIN3 or worse.

## Graphical abstract

What's new?

Cervical cancer screening programmes could benefit from an accurate triage test for human papillomavirus‐positive women based on self‐sampling. This epigenome‐wide association study examined a large number of cervical cancers and cervical intraepithelial neoplasias grade 3 (CIN3) compared to controls using the 850 k EPIC methylation array. The results identified DNA methylation at PAX1 and NREP‐AS1 as independent associated biomarkers for CIN3 or worse. Combined PAX1/NREP‐AS1 yielded a sensitivity of 83% and a specificity of 87%, and a mean AUC of 0.77 when validated in an independent CIN3 cohort, highlighting its potential for use in cervical screening programmes.

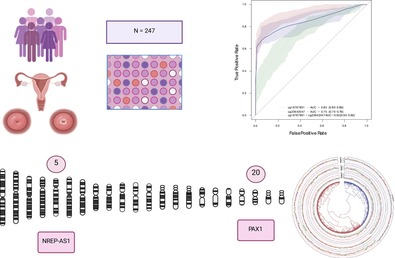

## 1 INTRODUCTION

Despite the advent of screening and vaccination, cervical cancer remains the fourth most common cancer in women globally. Most cervical cancers require persistent infection with high‐risk subtypes of human papillomavirus (hrHPV), making this a necessary, although not sufficient, condition for the development of invasive disease. Where implemented, cervical screening programmes have been highly successful in reducing the incidence and mortality from cervical cancer through monitoring and treatment of high‐grade cervical intraepithelial neoplasia (CIN). Traditionally, cervical screening programmes have relied on cytology of exfoliated cervical cells; however, more recently, hrHPV DNA testing has replaced cytology in many national screening programmes. The latter is anticipated to increase sensitivity and offer greater protection against invasive cancer^1^ but also to increase the number of women testing positive due to the decreased specificity of hrHPV testing. As such, further ‘triage’ markers are needed to increase the efficiency of screening. Host DNA methylation has been suggested as a triage test to identify women with high‐grade CIN or cervical cancer from those detected as hrHPV positive at screening.

DNA methylation represents a central gene expression regulatory process, and differences in methylation have been detected in multiple cancer types when compared to counterpart benign tissues.^2^, ^3^, ^4^ DNA methylation may reflect ongoing carcinogenesis through pathways such as loss of tumour suppressor gene function and other aberrant cell cycle and cellular growth controls. Although not necessarily causative, differential methylation at specific loci has been proposed as a potential measurable and stable marker of high‐grade CIN and cervical cancer.

Targeted studies comparing methylation levels in pre‐defined genes of cervical lesions versus normal controls have identified differential methylation in several candidate genes including *CADM1, MAL*, *and MiR124‐2*,^5^ *FAM19A4*,^6^ *POU4F3*,^7^ *EPB41L3*,^8^ *ZNF582*,^9^ *PAX1*, *SOX1*, and *SOX14*.^10^, ^11^, ^12^, ^13^ Subsequently, there has been evaluation of the performance of DNA methylation at specific loci as a diagnostic test to identify those with high‐grade disease. Some of the most researched tests include commercially developed assays such as QIAsure (Qiagen, Germany), a methylation test combining *FAM19A4* and *MiR124‐2* genes, and GynTect (Oncgnostics, Germany) (ASTN1, DLX1, ITG4, RXFP3, SOX17, and ZNF671). In an HPV positive screening population of 2384 women, QIAsure was reported to have a sensitivity (Se) of 77.2% and specificity (Sp) of 78.3%^14^ to detect CIN3. In another study of HPV positive women (*n* = 149) which compared the performance of QIAsure to GynTect, GynTect had a higher specificity (87.6% vs. 67.4%) for detecting both CIN2 or worse (CIN2+) and CIN3 or worse (CIN3+) (84.1% vs. 68.2%).^15^

Although the performance of methylation markers is promising, randomised clinical trials are still lacking, and comparisons to other potential triage tests are also limited. Meanwhile, the epigenome in cervical cancer has not been fully explored. Only one previous epigenome‐wide study (EWAS) has been performed on the EPIC array (Illumina 850 K),^16^ which included mainly preinvasive lesions and eight invasive cervical carcinomas (ICC). This study by El‐Zein et al.^16^ included 186 women attending colposcopy and examined liquid‐based cytology (LBC) cervical samples to identify four differentially methylated genes (*CA10*, *DPP10*, *FMN2*, and *HAS1*). Other EWAS have been limited to very small numbers; for example, Wang et al. studied 12 women with ICC to identify four genes: SLC6A5, SOX1, SOX14, and TBX20.^17^ Another EWAS included six ICC and 18 CIN3 samples and reported 24 potential biomarker genes.^18^ Siegal et al.^19^ identified a 36‐gene panel from 10 ICC on the Illumina 450 k array, while Boers et al.^20^ found eight candidate methylation markers for CIN2/3 (*ZSCAN1, ST6GALNAC5, ANKRD18CP, CDH6, GFRA1, GATA4, KCNIP4*, and *LHX8*). Due to the small numbers in these studies, it is highly likely that novel and more powerful methylation signatures exist for the effective identification of high‐grade cervical lesions and especially ICC.

The use of EWAS methodologies allows the full‐resolution exploration of genome‐wide epigenetic sites, including areas of the genome that have not been previously assessed in targeted studies, which therefore favors the potential for identifying novel markers. While the use of biomaterial from the target tissue—here cervical samples—may improve statistical power and aid interpretability, it may also increase and complexify the correlation structure across measurements of per‐site methylation levels. Using a conditional analysis framework, as widely used in GWAS studies,^21^ is key to identifying a sparse set of methylation sites, jointly associated with the outcome of interest. Identifying this reduced number of sentinel CpG sites is key to facilitating the translation of methylation markers to diagnostic testing in cervical screening programmes.

Our study represents, to the best of our knowledge, the largest EWAS to date comparing differential methylation in women with cervical cancer (ICC) or precancerous lesions of high malignant potential (CIN3) in comparison to benign controls, using cervical LBC samples.

## 2 METHODS

### 2.1 Study design

Our case–control study recruited participants prospectively between 2014 and 2020 from two affiliated university teaching hospitals in London (UK) and one university teaching hospital in Thessaloniki (Greece) as part of the PRECAC study^22^ biobank held at Imperial College London. Briefly, we included non‐pregnant women aged 18–70 years who attended colposcopy, gynaecology clinics, or accessed inpatient gynaecological services at Imperial College NHS Healthcare Trust (St Mary's Hospital 2016–2020, Hammersmith Hospital 2016–2020), or colposcopy clinics at the Aristotle University of Thessaloniki (2014–2019). Cases were defined as women with a histologically proven CIN3 or invasive carcinoma of the cervix, graded by a senior cytopathologist or gynaecological pathologist according to the Bethesda (cytology) and National Health Service Cervical Screening Programme (NHSSCP) systems (histopathology), and controls were defined as women with a histologically proven diagnosis of normal tissue (and/or no low‐ or high‐grade cytological abnormality) with no history of CIN or cervical cancer. All LBC samples were tested for hrHPV status using the Abbott RealTime HR HPV assay (14 HR‐HPR types) (Edinburgh, UK) and the Anyplex HPV 28 (28 low‐risk HPV and high‐risk HPV (hrHPV) types) (Edinburgh, UK). Detailed medical and gynaecological history was collected at recruitment via a self‐reported questionnaire. Women with previous cervical cone biopsy, other gynaecological cancers, HIV, or hepatitis B/C positivity, or with another active genital tract bacterial infection were excluded. Ethnicity was self‐reported as Caucasian, Asian, Black, or other. Cervical samples were taken by a cytobrush using the ThinPrep Preservcyt system (Hologic, Crawley, UK) and stored at 4°C until use. Study participants provided written informed consent. Recruitment continued until the study sample size was reached.

### 2.2 DNA extraction and quantification

DNA extractions and Quality Control were conducted in the same laboratory (Imperial College London, UK). Whole genomic DNA was extracted from 2 to 4 ml of the Preservcyt solution using a QIAmp DNA Mini kit (Qiagen, Crawley, UK) according to the manufacturer's instructions and eluted in DNA‐free distilled water. Samples were tested for genomic DNA quality and quantity using the Nanodrop spectrophotometer (Thermofischer Scientific, MA) and the PicoGreen protocol (Quant‐iT PicoGreen double stranded DNA [dsDNA] products Invitrogen). Where DNA quality was insufficient as measured by 260/280 and 260/230 ratios, samples were re‐extracted. From a total of 328 samples collected, 84 samples were excluded due to persistently inadequate DNA quality at the second extraction. The remaining 244 samples were concentrated or diluted to a standard concentration of 50 ng/μl prior to bisulphite conversion.

### 2.3 Bisulphite treatment and methylation array

Bisulphite conversion and methylation array processing were undertaken at the Oxford Genomics laboratory (Oxford, UK) where 500 ng of DNA were bisulphite‐converted with the EZ‐96 DNA Methylation‐Gold™ Kit (Zymo Research, Orange, CA) according to the manufacturer's protocol. Next, the Illumina Infinium Human Methylation 850 k (EPIC) array (San Diego, CA) was hybridised as per the manufacturer's protocol. Methylation testing was performed blinded to hrHPV status, cytology, and histology. This array measures DNA methylation at >850,000 cytosine positions across the human genome, particularly enriched for promoters and enhancers. Samples were randomised with respect to slide and position on methylation arrays to mitigate batch effects.

### 2.4 Derivation of normalised DNA methylation values and quality control

Raw data were analysed in the form of the Illumina Intensity Data (IDAT) files as input to an in‐house analysis pipeline in R as previously described.^23^ Pre‐processing included background subtraction and dye‐colour correction to account for the dye bias seen in Infinium II probes. This was done by equalising the intensities in the green and red channels to the average intensity across the two colours as measured by normalisation control probes present on the BeadChip. Samples failing quality control were identified by those lying 3 SD outside of the sample distribution for bisulphite converted probes, and by measuring the average signals along the three types of beads (type 1 green, type 1 red and type II); all samples passed this initial quality control step (Figure S1). DNA methylation‐inferred sex was evaluated via visualisation of the proportion of missing values on chromosome Y as a function of median methylation proportions on chromosome X; two samples were removed following the identification of two outlying women with lower proportions of missing data on chromosome Y (Figure S2). Following sample quality control, *N* = 241 samples remained. Probes with more than 30% missing values in more than (*N* = 22,248) were excluded. The methylation level at each Cytosine‐phosphate‐Guanine (CpG) was expressed as M‐value, which was obtained from logit_2_‐transformation of the fraction of methylated cytosines at that specific location (*β* value). To correct for technical variation, we used a series of linear mixed models with methylation M‐values for each of the CpG sites as the outcome, and chip and position as random intercepts. The residuals were extracted from these models and used as de‐noised methylation levels, as previously described.^24^ Standard packages to adjust for cell type in methylation analyses, for example, the Houseman approach,^25^ could not be applied as there was no reference data available for cell type distribution in cervical epithelial samples.

### 2.5 Epigenetic association analysis

To identify differentially methylated CpG sites in CIN3 or cervical cancer (ICC) cases compared to benign controls, we performed a series of logistic regression models (binary outcome being CIN3 and/or ICC) using each of the (de‐noised) methylation levels as predictors and adjusted for DNA quantity, hrHPV status, and age. We corrected for multiple testing using the Bonferroni procedure.

We conducted a series of conditional analyses to identify CpG sites that independently contributed to the prediction of CIN3 and/or ICC status. For this, we used an iterative procedure further adjusting the models on the CpG site showing the strongest association with the CIN3 or ICC outcome. This procedure was repeated until no significant association could be detected after correction for multiple testing. The CpG sites showing the strongest association at each iteration are considered independently associated with the cervical cancer outcomes. Similar to previous genome‐wide studies,^21^ we conducted a series of conditional analyses and report here for each CpG site the conditional *p*‐value (*p* ^cond^), which is defined as the *p*‐value obtained from the model adjusted on CpG sites detected in previous iterations, that is, the model in which the corresponding CpG site showed the strongest association.

We used a clustering approach to identify groups of CpG sites bringing redundant information to the discrimination between cases and controls. This was done by applying hierarchical clustering with complete linkage on the CpG sites significantly associated with the outcome. The absolute Pearson's correlation, conditioned on the disease status, was used as a similarity metric.

To account for disease heterogeneity and progression, we subsequently conducted stratified analyses comparing the methylation levels of participants with (i) CIN3 only or (ii) ICC only, to controls.

As sensitivity analyses, we performed the logistic models without adjusting for hrHPV status. To further investigate the influence of hrHPV on genome‐wide methylation in cases versus controls, we performed the same analysis in hrHPV‐positive women only. To identify CpG sites associated with hrHPV status, we performed a logistic regression of de‐noised methylation levels against hrHPV status, adjusting for age, DNA quantity, and restricting to controls only.

### 2.6 Quantifying the contribution to disease risk prediction

To quantify the ability of the identified CpG sites to discriminate cases from controls, we conducted a series of Receiver Operating Characteristic (ROC) analyses. To prevent over‐fitting, the logistic models were trained on 80% of the participants (training set) and performances were evaluated on the remaining 20% of the samples (test set). The proportions of cases and controls in the training and test sets were fixed to those observed in the full sample. The procedure was repeated over 1000 iterations for a more reliable evaluation of the model performance. We reported the mean, 5th and 95th percentiles of the sensitivity, specificity, and Area Under the Curve (AUC) to evaluate the clinical performance and extent of prediction of methylation markers for the detection of CIN3 or cervical cancer lesions. For independent validation, we conducted the same procedure using publicly available data from another study (accession number GSE143752^16^) of 42 CIN3 cases and 54 control cervical LBC samples sequenced on the Illumina EPIC array.

## 3 RESULTS

### 3.1 Participant characteristics

Characteristics of the included study population (*N* = 241) are summarised in Table 1. Overall prevalence of HPV was 62.8%, with 92.1% of the cases and 24.6% of the controls testing HPV positive. Mean age was similar across cases (40.5 years), CIN3 (33.1 years) and ICC (50 years).

**TABLE 1:** Summary statistics for included participants.

	Controls (*N* = 114)	CIN3 cases (*N* = 73)	ICC cases (*N* = 54)	*p*‐value
Age (years) + SD	40.45 (12.27)	33.12 (7.29)	49.68 (15.26)	0.8146
hrHPV infection				
No	86 (75.44%)	0 (0.00%)	10 (18.52%)	0.0001
Yes	28 (24.56%)	73 (100.00%)	44 (81.48%)	
Smoking status				
Never smokers	41 (35.96%)	33 (45.21%)	22 (40.74%)	0.1211
Ever smokers	7 (6.14%)	7 (9.59%)	5 (9.26%)	
Missing	66 (57.89%)	33 (45.21%)	27 (50.00%)	
DNA quantity + SD	1.52 (0.47)	1.67 (0.39)	2.00 (0.42)	0.0001
Blood contamination				
Moderate	89 (78.07%)	65 (89.04%)	28 (51.85%)	
Heavy	25 (21.93%)	8 (10.96%)	26 (48.15%)	0.4536

*Note*: *p*‐value calculated using Fisher's exact test (two‐tailed) or Student's *t*‐test between all cases versus controls.

Abbreviations: CIN3, cervical intraepithelial neoplasia grade 3; hrHPV, high‐risk genotype of human papillomavirus; ICC, invasive cervical cancer.

### 3.2 Epigenome‐wide association analyses

Of the 843,611 CpG sites included for analysis, *N* = 38,584 sites were differentially methylated in CIN3/ICC cases compared to controls in the models adjusted for age, DNA quantity, and technical confounders, after correction for multiple testing using the Bonferroni procedure.

**FIGURE 1:**
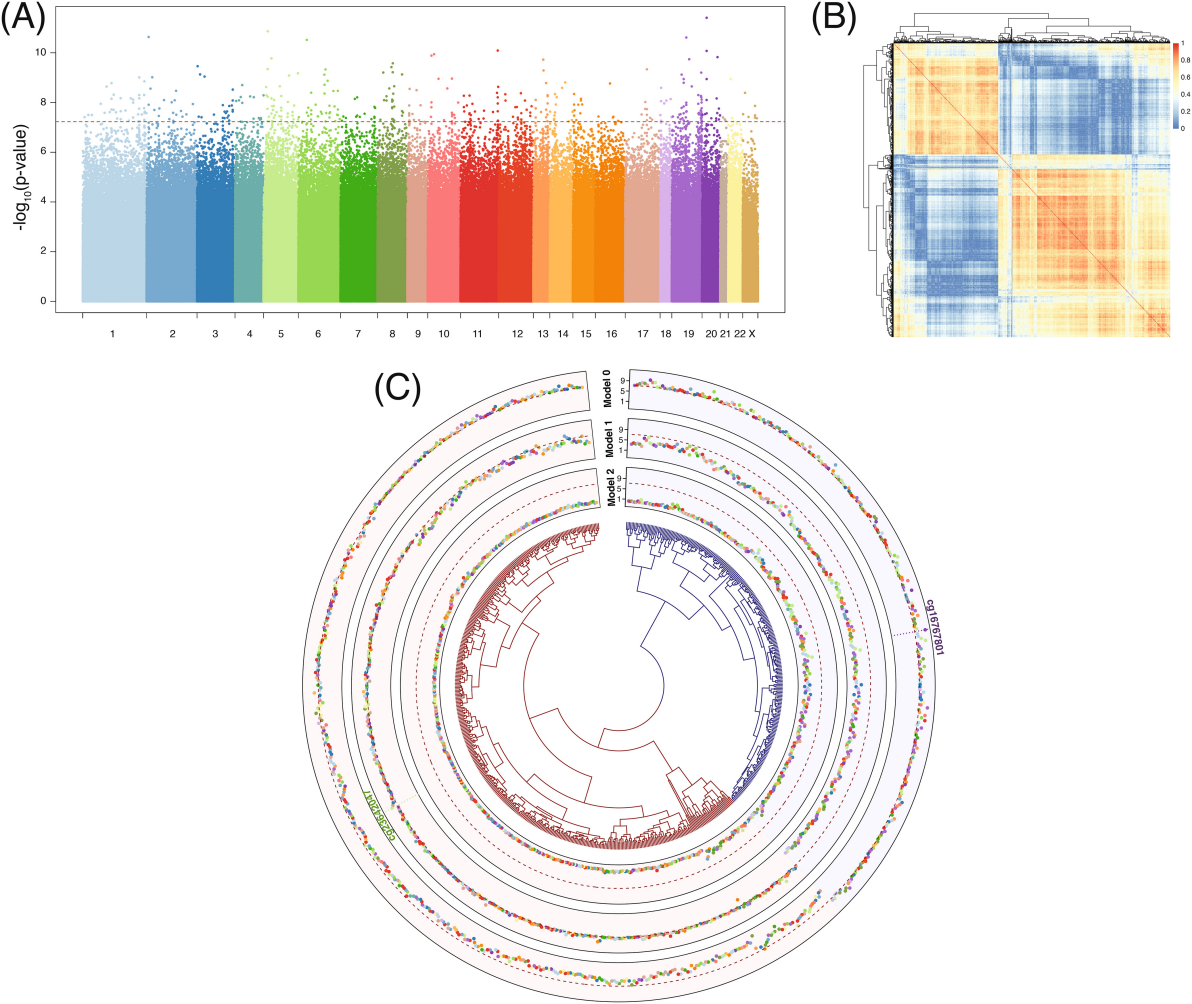
Associations between methylation levels and CIN3 (*N* = 73) or ICC (*N* = 54) status (vs. *N* = 114 controls) in the study population. (A) Manhattan plot showing the *p*‐values, measuring the strength of association, derived from logistic models adjusted for HPV status and represented on the‐log10 scale (Y‐axis). CpG sites (*N* = 843,611) are ordered by their position on the genome (X‐axis). The epigenome‐wide significance level is set to the Bonferroni‐corrected threshold (horizontal red line). (B) Heatmap of absolute correlations between the CpG sites (*N* = 727) significantly associated with CIN3 or ICC status in the model adjusted on HPV status, or further adjusted on cg16767801. To avoid spurious correlations, these are adjusted on the case/control status. CpG sites are ordered by hierarchical clustering with complete linkage using the absolute correlation as a similarity metric. (C) Circos plot showing the *p*‐values from conditional analyses on‐log10 scale. Models are adjusted on HPV status (Model 0), and further adjusted on cg16767801 (Model 1), and cg23642047 (Model 2). CpG sites (*N* = 727) are ordered based on the same hierarchical clustering. Lead CpG sites from each of the models are indicated with vertical dotted lines. Points are coloured by chromosome as in panel (A).

When adjusting for HPV status, the strength of the associations were attenuated (Table 2 and Figure S3). We identified *N* = 409 CpG sites with differentially methylated levels at the Bonferroni‐corrected significance level (Figure 1A and Table S1), which were spread across the whole genome. These CpG sites were also strongly associated with the ICC/CIN3 outcome in the model not adjusted for HPV status (*p*‐value <2.41 × 10^−7^).

**TABLE 2:** Top CpG sites in the main epigenome‐wide association analysis, ordered by *p*‐value in the logistic regression and restricted to a percentage methylation change of >20% between cases (CIN3 and Cancer) versus benign controls (*N* = 241).

CpG	Chr	Gene	OR	OR lower 95% CI	OR upper 95% CI	SE	*p*‐value	Δ beta
cg11358689	11	NTM	5.40	2.93	9.97	0.31	8.13E‐11	24.88%
cg21183256	8		5.23	2.82	9.73	0.32	2.65E‐10	24.80%
cg13368756	5	CTNND2	5.86	2.87	12.00	0.37	4.52E‐10	20.69%
cg01758512	6	FUT9	4.27	2.54	7.20	0.27	4.68E‐10	20.68%
cg02608002	19	BST2;MVB12A;BISPR	0.21	0.12	0.38	0.30	1.61E‐09	−20.92%
cg18397073	19	POU2F2	7.40	3.16	17.33	0.43	2.25E‐09	21.14%
cg23214755	2	E2F6	0.22	0.13	0.39	0.29	2.76E‐09	−26.60%
cg26047066	1		5.46	2.76	10.77	0.35	4.99E‐09	24.75%
cg18375860	1	RYR2	4.48	2.49	8.06	0.30	5.00E‐09	21.09%
cg04996219	5	CTNND2	4.81	2.51	9.21	0.33	5.88E‐09	22.39%
cg14035860	14		4.88	2.58	9.25	0.33	6.14E‐09	21.50%
cg15617814	11	NTM	4.45	2.44	8.09	0.31	6.20E‐09	20.74%
cg25001102	1		5.04	2.59	9.80	0.34	6.77E‐09	22.19%
cg08272731	1	LHX8	4.45	2.47	8.05	0.30	7.05E‐09	22.53%
cg03502002	18	GALR1	4.96	2.58	9.55	0.33	7.25E‐09	20.18%
cg00688962	4	KCNIP4	3.79	2.28	6.28	0.26	1.03E‐08	20.57%
cg01696784	2	SH3BP4	4.65	2.48	8.71	0.32	1.15E‐08	23.89%
cg24211976	15		4.81	2.59	8.94	0.32	1.20E‐08	29.64%
cg12492087	15	ZFP106	4.34	2.41	7.80	0.30	1.22E‐08	22.84%
cg08396985	2		4.56	2.47	8.42	0.31	1.23E‐08	27.72%
cg01972979	X	MGC16121;MIR503	4.53	2.47	8.31	0.31	1.37E‐08	20.11%
cg22370295	1	RYR2	4.41	2.43	7.99	0.30	1.69E‐08	20.80%
cg27607773	2	RBKS	4.32	2.42	7.72	0.30	1.99E‐08	21.52%
cg25095032	6	DDAH2	5.36	2.63	10.90	0.36	2.06E‐08	21.16%
cg19717586	11	NTM	4.33	2.35	7.95	0.31	2.26E‐08	25.33%
cg12785694	3	SMC4;MIR15B;MIR16‐2	0.24	0.14	0.43	0.29	2.28E‐08	−20.56%
cg10982443	14	KLHL33	5.04	2.54	9.99	0.35	2.33E‐08	23.49%
cg13849378	1	RGS7	4.08	2.26	7.38	0.30	2.62E‐08	21.03%
cg09632273	6	DDAH2	4.45	2.40	8.25	0.31	2.97E‐08	21.21%
cg19632836	13		4.01	2.28	7.06	0.29	3.06E‐08	20.07%
cg02763101	19	ZNF582	5.15	2.41	10.97	0.39	3.09E‐08	21.50%
cg24348495	20	TCEA2	4.58	2.45	8.59	0.32	3.32E‐08	21.23%
cg06330323	16	TSC2	4.95	2.46	9.96	0.36	3.67E‐08	23.04%
cg26609631	13	GSX1	3.79	2.19	6.56	0.28	4.02E‐08	21.51%
cg21660452	11	NRXN2	5.19	2.62	10.30	0.35	4.60E‐08	20.48%
cg23878255	7	CLEC5A	0.27	0.16	0.46	0.27	4.66E‐08	−20.64%
cg21130374	21	MX2	0.26	0.15	0.45	0.27	4.75E‐08	−20.94%
cg16020747	8	RALYL	3.67	2.17	6.21	0.27	5.26E‐08	26.21%
cg09515921	1	HLX	4.33	2.36	7.96	0.31	5.63E‐08	22.63%

*Note*: *Where intragenic, the gene is annotated from the UCSC reference gene browser. Intronic and intergenic CpG sites are annotated to genomic positions only.

Abbreviations: Chr, Chromosome; CI, confidence interval; OR, odds ratio of beta value; SE, standard error, Δ, change in % methylation value between cases and controls.

### 3.3 Conditional analyses

Conditional analyses adjusted on HPV status identified a set of two CpG sites bringing complementary information discriminating CIN3/ICC cases from controls. These were located in the (i) first exon of *PAX1* on chromosome 20 (cg16767801; OR = 6.32[95%CI = 3.20–12.50]; *p* = 3.90 × 10^−12^; Δ beta =16.4%) and (ii) gene body of NREP‐AS1 (cg23642047; OR = 0.33[95%CI = 0.21–0.52]; *p* = 3.34 × 10^−7^; Δ beta = −15.9%) on chromosome 5 (Table 3).

**TABLE 3:** Differentially methylated CpG sites identified as independent from conditional and clustering analyses in CIN3 and ICC.

CpG	Chr	Gene	Annotation	*N*	OR (95%CI)	*p* (*p*_cond_)	Δ beta
cg16767801	20	*PAX1*	First Exon	241	6.32 (3.20–12.5)	3.90 × 10^−12^ (3.90 × 10^−12^)	16.4%
cg23642047	5	NREP‐AS1	Gene Body	241	0.33 (0.21–0.52)	3.34 × 10^−7^ (2.45 × 10^−10^)	−15.9%

Abbreviations: Chr: chromosome; *N*: number of women included: OR: odds ratio of beta value; *p*: *p*‐value; *p*_cond_: conditional *p*‐value; Δ: change in % methylation value between cases and controls.

In the baseline model (model 0) we identified *N* = 409 differentially methylated CpG sites between CIN3/ICC cases and benign controls. Of these, cg16767801 showed the strongest association (*p* = 3.90 × 10^−12^). When adjusting for the methylation level at cg16767801 in the conditional analysis, a total of *N* = 318 CpG sites that were not identified in model 0 were identified to be differentially methylated. Using hierarchical clustering, we identified two clusters of strongly correlated CpG sites: one proxied by cg16767801 as sentinel (in blue) and one proxied by cg23642047 (in red) (Figure 1B,C). Figure 1C shows that the *p*‐values of CpG sites from the cluster with cg16767801 (or cg23642047) are strongly attenuated upon adjustment for the corresponding lead CpG site. None of the associations continue to be significant after correction for multiple testing when adjusting for both cg161767801 and cg23642047 (model 2).

### 3.4 Stratified analyses of preinvasive (CIN3) versus invasive phenotypes

When stratifying the analysis into CIN3 or ICC phenotypes, we observe a much lower number of associations for CIN3 than ICC (*N* = 62 differentially methylated CpG sites at Bonferroni‐corrected significance level, Figure S4). A total of *N* = 5232 CpG sites were found differentially methylated between ICC cases and controls in logistic models adjusted for hrHPV status. The two CpG sites identified in conditional analyses were strongly associated with ICC status (*p*‐value of 4.34 × 10^−12^ for cg16767801 and 9.74 × 10^−7^ for cg23642047), while only cg16767801 was found differentially methylated in CIN3 cases compared to controls.

### 3.5 Sensitivity analyses

Despite the smaller sample size (*N* = 111 cases and *N* = 25 controls), a total of *N* = 719 CpG sites were found differentially methylated in hrHPV‐positive cases compared to hrHPV‐positive controls. The results were consistent with those from the models adjusted for HPV status in the full population (*p*‐values of <1.68 × 10^−3^ in the analyses restricted to hrHPV positives for the *N* = 409 statistically significant CpG sites identified in the full population) (Figure S5).

Due to the high degree of missing data on smoking, we could not include smoking status in the final model; however, there was no significant difference in the proportion of known current or ever smokers between cases and controls (Table 1).

### 3.6 Diagnostic accuracy

**FIGURE 2:**
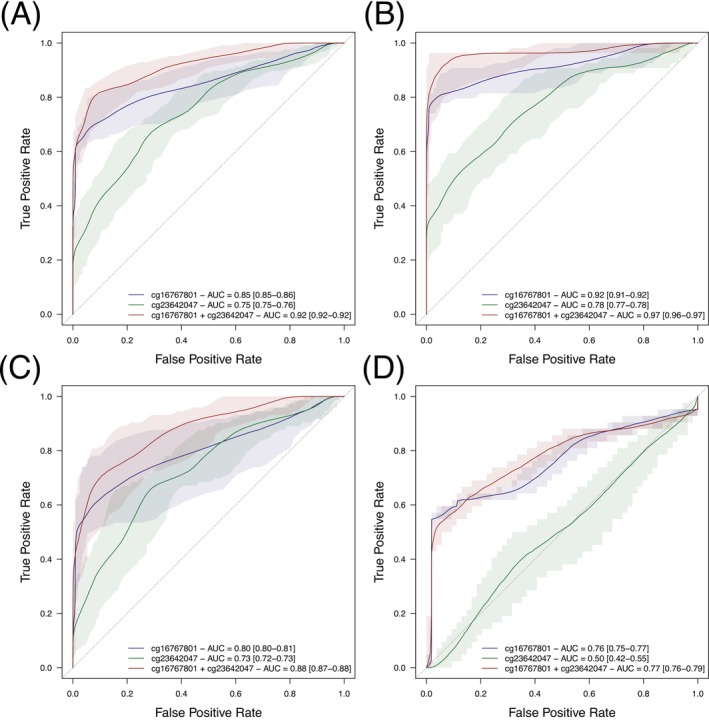
Receiver operator curves (ROC) for logistic models including cg16767801 (in blue), or cg23642047 (in green) or both (in red) as predictors and all cases (A), cancer (B) or CIN3 (C, D) as outcome. Models were fitted on 1000 training sets with 80% of the cases and 80% of the controls and performances were evaluated on a test set with the remaining 20% of participants. The pointwise average, 5th and 95th percentiles of the True and False Positive Rates and Area Under the Curve (AUC) are reported. Results presented in panels A, B and C were obtained using our study population (*N* = 114 controls, *N* = 73 CIN3 cases and *N* = 54 cancer cases). An independent validation set (accession number GSE14375s, with *N* = 54 controls and *N* = 42 CIN3 cases) was used in panel D.

Prediction models based on the methylation levels of a single CpG site yielded very good discrimination performances between CIN3/ICC cases and controls and a mean AUC of 0.85 (95% CI 0.85–0.86) for cg16767801, and an AUC of 0.75 (95% CI 0.75–0.76) for cg23642047 (Figure 2A). The performances were further improved when using both CpG sites as predictors with an average AUC of 0.92 (95% CI 0.92–0.92) with a corresponding sensitivity of 83% (95% CI 83%–83%) and specificity of 87% (95% CI 87%–88%). Irrespective of the number of CpG sites considered, prediction performances of the bigenic model were better for cervical cancer cases (mean AUC of 0.97 [95% CI 0.96–0.97]; Se = 96% [95% CI 96%–96%]; Sp = 72% [95% CI 72%–72%]) than for CIN3 cases (mean AUC of 0.88 [95% CI 0.87–0.88]; Se = 83% [95% CI 82%–84%]; Sp = 71% [95% CI 71%–72%]) (Figure 2B,C and Table S2). Further assessment of the prediction performances of these two CpG sites in an independent validation set (*N* = 54 controls and *N* = 42 CIN3 cases) (Figure 2D) revealed a sensitivity of 73% (95%CI 69%–76%), a corresponding specificity of 68% (95%CI 65%–70%), and a mean AUC of 0.77 (95% CI 0.76–0.79) using the two CpG sites as predictors. The contribution of cg23642047 to the prediction of CIN3 cases in the validation set was very limited and yielded an increase in mean AUC <1% over that from cg16767801.

## 4 DISCUSSION

Using an epigenome‐wide approach, we identified 409 differentially methylated CpG sites in cervical cancer or CIN3 patients. Conditional analyses combined with clustering analyses identified two clear groups of CpG sites, those relating to cg16767801 (*PAX1*) and those relating to cg23642047 (*NREP‐AS1*). None of the CpG sites within each of the clusters was found associated with the cancer/CIN3 outcome while adjusting for the sentinel CpG site, suggesting that these sentinel markers accurately summarized the disease‐relevant information of the 409 CpG sites. We thoroughly explored for confounding by HPV status and adjusted for HPV positivity in the main analysis. As a bigenic methylation marker, *PAX1/NREP‐AS1* (cg16767801 + cg23642047) was able to detect CIN3 or invasive cervical cancer with a sensitivity of 83%, specificity of 87%, and an AUC of 0.92 in a validation set, with a good predictive performance for CIN3 in an independent replication set.

To our knowledge, this is the first and largest study to explore the differential methylation in cervical cancer and CIN3 samples versus benign cervical samples using an epigenome‐wide approach. Previous studies have been limited to primarily pre‐invasive samples^16^, ^26^ given the heterogenous nature of CIN2, where over half of CIN2 may spontaneously regress,^27^ in this study, we chose to include only cancer and CIN3 as cases, facilitating identification of markers that associate with true preinvasive lesions. Our analysis included over 50 invasive cervical cancer samples and expanded coverage through the Illumina 850 k array, which, although it is enriched for promoters and enhancers, includes more gene body CpG sites than other arrays. We observed that differentially methylated regions were scattered widely across the whole genome, and both hypermethylation and hypomethylation are present in high‐grade cervical lesions, consistent with previous studies.^28^, ^29^

The functional effects of CpG methylation are important for both understanding cervical carcinogenesis and the biological plausibility of the biomarker. *PAX1* (paired box gene 1) forms a member of nine well‐characterised transcription factors important primarily for tissue development and cellular differentiation. As a group, they are reported to be frequently expressed in cancer and play a necessary role in cancer cell survival,^30^ the silencing of *PAX* genes having been associated with apoptosis in cancer cell lines, while endogenous *PAX* gene expression is thought to be required for the growth and survival of cancer cells.^30^ Inheritance of *PAX8* genomic mutations has been previously associated with cervical cancer by our team.^21^ While mechanisms are incompletely understood, the loss of *PAX1* function through DNA methylation may promote carcinogenesis through the disruption of kinase and phosphatase interaction in the cervical epithelium.^31^

*PAX1* methylation has been previously associated with the detection of cervical preinvasive and invasive disease in targeted studies of *PAX* genes, performed largely in East Asian populations,^32^, ^33^, ^34^ but not in previous EWAS. In a 2015 meta‐analysis^35^ which pooled the diagnostic test accuracy estimates for methylation across various CpG sites in *PAX1*, sensitivity for the detection of CIN3 (*N =* 1385; 7 studies) was 77% (95%CI 58–89%) and specificity was 92% (95%CI 88%–94%). While in a more recent meta‐analysis, Kelly et al. report a sensitivity of 68.4% (95%CI 53%–81%) and specificity of 84% (95% CI 74%–90%) for *PAX1* markers (*N* = 1217; 5 studies).^36^ Our conditional and clustering analyses demonstrated that the addition of *NREP‐AS1* methylation appears to independently improve the overall accuracy of *PAX1* for the detection of cervical cancer. As a bigenic marker, *PAX1/NREP‐AS1* (cg16767801 + cg23642047) was able to detect CIN3 or ICC with a sensitivity of 83% and specificity of 87%. To our knowledge, the association of *NREP‐AS1* methylation with cervical cancer is novel and has not been previously described.

The best triage strategy to detect high‐grade CIN and cancer in hrHPV women is not yet clear and practice varies greatly by country, with most screening programs opting for reflex cytology or HPV 16/18 genotyping. To evaluate the potential for clinical translation of the identified markers as diagnostic triage tests, larger studies in independent screening populations of HPV‐positive women are needed. Direct comparisons of methylation markers to other triage tests are still limited, but cytology is the most studied comparator test. In a cross‐sectional analysis, QIAsure (*FAM19A4/mir124‐2*) was compared to cytology in a screening cohort of HPV‐positive women; sensitivity to detect CIN3+ was reportedly similar to cytology (71.3% vs. 76.0%) but with a lower specificity (78.3% vs. 87.0%). In another case–control study of HPV‐positive women, triage by the S5 classifier (UK) methylation test (*EPB41L3/*HPV16/18/31/33 L1 and L2 genes) was compared to triage by cytology plus HPV 16/18 genotyping. Relative sensitivity of the S5 classifier for detection of CIN3 was higher than combined abnormal cytology plus HPV 16/18+ (93.2% vs. 86.4%) but with a lower corresponding positive predictive value (PPV) (18.2% vs. 19.3%) and specificity (41.8% vs. 49.8%), however this did not reach statistical significance.^37^ Kocsis et al.^7^ compared *POU4F3* methylation to cytology in over 1200 HPV positive women and reported a higher sensitivity (70.1% vs. 42.7%) with a similar specificity (81.4% vs. 80.4%) for CIN2+.^14^ Chang et al.^38^ compared *PAX1* methylation to cytology and HPV 16/18 genotyping in a Taiwanese screening population of 429 hrHPV positive women. *PAX1* methylation, cytology and HPV16/18 genotyping all had a similar sensitivity (79.3% vs. 78.6% vs. 79.3%) but *PAX1* and cytology had higher specificity (92.5%, 95.7%) than HPV 16/18 genotyping (76.8%) for CIN3+. While in a Dutch population of 739 hrHPV positive women, sensitivity for *ASCL1*/*LHX8* methylation for detection of CIN3+ was similar (76.9%) to HPV 16/18 genotyping (73.5%) and lower than cytology (89%) however, this did not reach statistical significance, likely because case numbers were small (*N* = 91). Specificity was higher for *ASCL1*/*LHX8* methylation (74.5%) than HPV 16/18 genotyping (67.5%) and cytology (62.3%). For CIN2+ as an outcome, sensitivity was lower for *ASCL1/LHX8* methylation vs. cytology (59.5% vs. 84.7%) while specificity was slightly higher (76.1% vs. 67.8%). The evaluation of methylation markers restricted to screening populations of HPV‐positive women, which includes direct comparisons to other triage markers, as well as evaluation of cut‐offs to balance sensitivity and specificity, is now needed to quantify the utility of methylation markers, including those we highlighted in the present study, in population screening programs.

As previously described, several methylation markers have reached the stage of commercial development. In our analysis, *ZNF582* and *EPB41L3* (S5 classifier) genes, which are both found in commercially available tests, did replicate, while genes found in GynTect (*ASTN1, DLX1, ITGA4, RXFP3, SOX17*, and *ZNF671*) and QIAsure (*FAM19A4* and *miR124‐2*) did not. Several genes reported as the top signals in a previous EWAS on the EPIC array also replicated, including *ATP10A, FMN2, CA10, RALYL, SOX1, NTM, KCNIP4, CLVS2, SOX11*, and *MDGA2* (Table S3).

Sensitivity and specificity of *PAX1/NREP‐AS1* were higher for the detection of ICC than CIN3 (CIN3 Se = 83% Sp = 71%; ICC Se = 96% Sp = 72%). CIN3 signals were found to be much attenuated across the genome when compared to invasive cancer, despite the larger sample size. This is consistent with previous epigenome‐wide studies where greater deltas were observed between cancer versus normal tissue than CIN3 versus normal tissue.^16^, ^18^ One study which investigated *ATP10A* and *HAS1* as a single marker^16^ reported a perfect prediction of ICC (Se = 100%; AUC 1.00) but with a large drop in sensitivity and specificity (68% and 60%, respectively) for CIN3. In an independent comparative analysis,^15^ the sensitivity of GynTect for CIN3 was 60%, and 100% for ICC (*N* = 123), while in the same study the sensitivity of QIAsure was 74.3% for CIN3 and 98.3% for ICC (*N* = 519).^15^ It is known that more global and aberrant methylation processes occur in invasive than in preinvasive lesions, particularly promoter hypermethylation of tumour suppressor genes.^39^ Additionally, high‐grade CIN lesions represent a heterogeneous stage of disease, where only a subset will progress to cancer over a period of 15–30 years. Study of longitudinal samples of CIN2 that progressed versus regressed also suggests the heterogeneous nature of these lesions.^40^, ^41^ Further exploration of methylation patterns in CIN3 compared to ICC is therefore warranted to understand the potential of methylation to differentiate novel cervical lesion subtypes, and additionally the clinical applications of diagnostic methylation tests for the detection of CIN3 as well as invasive lesions.^42^

Adjustment for HPV status attenuated methylation signals across the genome. We further investigated the HPV effect by restricting it to HPV‐positive women, whereby comparing beta coefficients and *p*‐values to the HPV‐adjusted analysis, we confirmed the importance of HPV status in the association between methylation and cervical disease. To our knowledge, this has not been previously described in genome‐wide studies. However, it has been previously demonstrated that HPV infection status alters methylation patterns.

## 5 LIMITATIONS

The self‐reported data on smoking was incomplete, and this could not be accounted for in the model, which is a limitation of this study. As smoking is known to affect methylation, this may have introduced bias; however, the number of smokers was well balanced across cases and controls, which should minimise any confounding. Additionally, contamination of the sample with other cell types could not be adjusted for. It is common to adjust for blood cell distribution within epigenetic studies based on serum samples; however, as there is no reference for cervical epithelial cells, this could not be accounted for. We adjusted our analyses for DNA quantity; however, to reflect the additional DNA obtained from samples taken from cancerous lesions compared to benign cervices. DNA quantity and methylation levels were very stable in cervical samples collected as early as 2014, suggesting good applicability to self‐sampling. Our case–control study facilitated the identification of two independent sentinel CpG sites with differential methylation in relation to case status. These may point toward two separate pathways involved in pathogenesis. However, for the methylation of these sites to be rigorously assessed as diagnostic markers for the triage of HPV‐positive women, prospective studies are warrented, particularly in consecutively recruited patients representing a screening population, including head‐to‐head trials against other potential triage markers.

## 6 CONCLUSIONS

In this study, we use a hypothesis‐free epigenome‐wide approach to identify human methylation signals that are associated with CIN3 and cervical cancer. We detected two mutually independent human methylation markers with an AUC of 0.77 in an external validation cohort of CIN3. The addition of *NREP‐AS1* adds additional value to *PAX1* as a marker, particularly for invasive lesions. Host methylation signals appear to be a stable marker of high‐grade disease and have the potential to improve the accuracy of cervical screening through identifying truly progressive lesions from transient HPV infections; however, robust prospective trials of methylation markers in HPV‐positive screening populations are now urgently needed to fully understand the diagnostic accuracy and clinical translation.

## AUTHOR CONTRIBUTIONS

**Sarah J. Bowden:** Conceptualization; methodology; formal analysis; investigation; data curation; funding acquisition; writing – original draft; writing – review and editing. **Barbara Bodinier:** Formal analysis; writing – review and editing; data curation; investigation. **Maria Paraskevaidi:** Investigation; writing – review and editing. **Ilkka Kalliala:** Investigation; writing – review and editing. **Maria Nasioutziki:** Investigation; data curation; writing – review and editing. **Laura Burney Ellis:** Writing – review and editing. **Ruben Colindres Zuehlke:** Formal analysis; writing – review and editing. **James M. Flanagan:** Formal analysis; methodology; writing – review and editing; writing – original draft. **Maria Kyrgiou:** Conceptualization; methodology; investigation; supervision; funding acquisition; writing – review and editing; writing – original draft. **Marc Chadeau‐Hyam:** Methodology; formal analysis; writing – review and editing; supervision; writing – original draft.

## FUNDING INFORMATION

Sarah J. Bowden, Laura Burney Ellis, and Maria Kyrgiou acknowledge support from Imperial NIHR BRC and the Wellcome Trust (grant No. P77712) and Imperial Health Charity (grant No. P73337; grant No. RFPR2223_27). Maria Paraskevaidi acknowledges support from Cancer Research UK (P92114). Marc Chadeau‐Hyam and Barbara Bodinier acknowledge support from Cancer Research UK, Population Research Committee Project grant ‘Mechanomics’ (grant No. 22184 to Marc Chadeau‐Hyam). Marc Chadeau‐Hyam acknowledges support from the H2020‐EXPANSE (Horizon 2020 grant No. 874627) and H2020‐LongITools (Horizon 2020 grant No. 874739). James M. Flanagan acknowledges support from Ovarian Cancer Action Risk and Prevention programme and the Imperial Cancer Research UK Centre.

## CONFLICT OF INTEREST STATEMENT

MC‐H holds shares in the O‐SMOSE company. Consulting activities conducted by the company are independent of the present study. All other authors have no competing interests to declare.

## ETHICS STATEMENT

Ethical approval was obtained from the National Research Ethics Service Committee London—Fulham (approval number 13/LO/0126) and the Aristotle University of Thessaloniki Bioethics Committee (approval number 123/08‐12‐2014). Informed consent was obtained from all individual patients included in the study.

## Supporting information


**DATA S1.** Supporting Information.

## Data Availability

The raw Illumina HumanMethylationEPIC BeadChip array data and corresponding metadata have been uploaded to the Gene Expression Omnibus (GEO accession number GSE287994). Further information is available from the corresponding author upon request.
