# Supplementary material for: DNA methylation signatures of cervical pre‐invasive and invasive disease: An epigenome‐wide association study
Source: Int J Cancer. 2025 Mar 10;157(2):305–16. doi: 10.1002/ijc.35406 (PMC12079623; doi:10.1002/ijc.35406)
Supplement: Supplementary file 1 — DATA S1. Supporting Information. [file IJC-157-305-s001.pdf]

SUPPLEMENTARY MATERIAL

Title: DNA METHYLATION SIGNATURES OF CERVICAL PRE-INVASIVE AND INVASIVE DISEASE: AN EPIGENOME-WIDE ASSOCIATION STUDY

Sarah J Bowden[1-3] PhD, Barbara Bodinier[4] PhD, Maria Paraskevaidi[1] PhD, Ilkka Kalliala PhD [1,5], Maria Nasioutziki MD PhD[6], Laura Burney Ellis[1-3] MBChB, Ruben Colindres Zuehlke[4] MSc, James M Flanagan[2]* Prof, Maria Kyrgiou[1-3]* Prof, Marc Chadeau-Hyam[4]* Prof.

Table of contents:

1. **Supplementary Table 1.** Summary results for 409 CpG sites Bonferroni significant in main EWAS analysis, adjusted for HPV status
2. **Supplementary Table 2**. Prediction performance as measured by the sensitivity and specificity for logistic models including cg16767801 and/or cg23642047 as predictor(s) and comparing controls (N=114) to all cases (CIN3 and Cancer) (A, N=127), CIN3 cases only (B, N=73) or cancer cases only (C, N=54). Performance metrics are reported as the mean, 5th and 95th quantiles computed over N=1,000 subsamples for different proportions of the population predicted as cases.
3. **Supplementary Table 3:** Replication of commercial or research methylation assays or previously published genome-wide studies
4. **Supplementary Figure 1.** Visualisation of technical metrics for Illumina 850K probes. Samples are represented along three variables measuring the average signals along the three types of beads (type I green, type I red and type II). No outlying observations were observed.
5. **Supplementary Figure 2.** Visualisation of the proportion of missing values on chromosome Y as a function of median methylation proportions on chromosome X. Women are expected to show higher proportions of missing values on chromosome Y and higher median proportions on chromosome X compared to Men.
6. **Supplementary Figure 3:** Association between CpG sites and CIN3 (N=73) or ICC (N=54) status (N=114 controls) unadjusted versus adjusted for HPV status in the study population. (A)Manhattan plot showing the P-values, measuring the strength of association, derived from logistic models **unadjusted for HPV status** and represented on the -log10 scale (Y-axis). CpG sites (N=843,611) are ordered by their position on the genome (X-axis). (B) Volcano plot demonstrating distribution of methylation beta-values by -log10(P-value) unadjusted for HPV status where 0 represents the null value and the red dashed line represents the Bonferroni-corrected EWAS significance level. Genome-wide a greater gain in methylation is observed in association with CIN3 or ICC case status across the assayed CpG sites on the EPIC array. (C) Manhattan plot showing the P-values, measuring the strength of association, derived from logistic models **adjusted for HPV status** and represented on the -log10 scale (Y-axis). CpG sites (N=843,611) are ordered by their position on the genome (X-axis). The epigenome-wide significance level is set to the Bonferroni-corrected threshold (horizontal red line)

7. **Supplementary Figure 4.** Manhattan plot showing the P-values, measuring the strength of association, derived from logistic models adjusted for HPV status and represented on the -log10 scale (Y-axis) for CIN3 only (A) and ICC only (B). CpG sites (*N*=843,611) are ordered by their position on the genome (X-axis). The epigenome-wide significance level is set to the Bonferroni-corrected threshold (horizontal red line). Scatter plots of beta values (C, left) and -log10(P-value) (C, right) for all CpG sites identifies as statistically significant in the main analysis (N=409), are plotted separately for CIN3 (y-axis) and ICC (X-axis)
8. **Supplementary Figure 5:** Scatter plots showing the correlation of beta methylation values (left) and -log10(P-value) (right) for Bonferroni significant CpG sites (N=409 CpG) from logistic models of the main analysis with the CIN3/ICC status as the outcome adjusted for HPV status (X-axis, N=114 controls and N=127 cases) or restricted to HPV positive participants (Y-axis, N=25 controls and N=111 cases)

SUPPLEMENTARY TABLES

Supplementary Table 1. Summary results for 409 CpG sites Bonferroni significant in main EWAS analysis, adjusted for HPV status

CpG	CHR	BP	GENE	OR	OR 95%CI lower	OR 95%CI upper	COEF	SE	P Value	delta
cg16767801	20	21686548	PAX1	6.32	3.20	12.50	1.84	0.35	3.9009E-12	16.39%
cg07195011	5	11904114	CTNND2	7.05	3.25	15.30	1.95	0.40	1.3655E-11	18.38%
cg17213402	2	5813650		5.80	3.04	11.08	1.76	0.33	2.3096E-11	19.48%
cg27058257	19	30019529	VSTM2B	13.84	4.08	46.93	2.63	0.62	2.425E-11	18.77%
cg22085751	6	28921976		5.76	3.05	10.88	1.75	0.32	3.0224E-11	12.48%
cg11358689	11	131781260	NTM	5.40	2.93	9.97	1.69	0.31	8.1265E-11	24.88%
cg09252495	20	21503898		7.63	3.30	17.65	2.03	0.43	8.4004E-11	13.74%
cg00530925	10	23463094		7.66	3.42	17.15	2.04	0.41	1.1404E-10	13.13%
cg12178980	10	11206870	CUGBP2	14.77	4.14	52.72	2.69	0.65	1.3004E-10	9.33%
cg02409888	20	58568926	CDH26	6.68	3.33	13.40	1.90	0.35	1.496E-10	10.62%
cg09499856	5	41510325	PLCXD3	6.50	3.16	13.37	1.87	0.37	1.6784E-10	7.88%
cg14587524	19	38183262	ZNF781	6.77	3.17	14.48	1.91	0.39	1.819E-10	19.01%
cg09083627	13	88324597	SLITRK5	6.45	3.05	13.63	1.86	0.38	1.884E-10	13.91%
cg21183256	8	73163868		5.23	2.82	9.73	1.66	0.32	2.6508E-10	24.80%
cg07351192	3	5137773		7.53	3.37	16.81	2.02	0.41	3.4555E-10	12.11%
cg25260137	8	65282185		4.66	2.62	8.31	1.54	0.29	3.7587E-10	19.47%
cg13368756	5	11904127	CTNND2	5.86	2.87	12.00	1.77	0.37	4.5191E-10	20.69%
cg02688304	17	50236104	CA10	5.47	2.84	10.56	1.70	0.34	4.6009E-10	18.20%
cg01758512	6	96463902	FUT9	4.27	2.54	7.20	1.45	0.27	4.676E-10	20.68%
cg12989574	13	93879544	GPC6	9.27	3.53	24.34	2.23	0.49	5.1297E-10	11.48%
cg25832771	8	72756058	MSC	6.37	2.94	13.79	1.85	0.39	6.0599E-10	16.78%
cg13093774	8	22089400	PHYHIP	7.59	3.28	17.54	2.03	0.43	6.2214E-10	13.46%
cg09555914	19	58011309	ZNF773	11.52	3.60	36.83	2.44	0.59	6.2696E-10	13.37%

cg17403609	5	180075862	FLT4	7.66	3.31	17.74	2.04	0.43	6.6339E-10	15.26%
cg13912117	8	132054555		5.41	2.81	10.44	1.69	0.34	7.4124E-10	15.84%
cg19112357	3	13834712		6.48	3.15	13.31	1.87	0.37	7.4163E-10	14.87%
cg17509967	19	13617094	CACNA1A	7.31	3.15	16.97	1.99	0.43	7.5355E-10	15.92%
cg17644557	6	86159103	NT5E	6.70	3.15	14.24	1.90	0.38	7.6121E-10	11.54%
cg14739859	5	145758710		4.73	2.60	8.60	1.55	0.30	8.2525E-10	8.22%
cg07704959	3	40471662		4.36	2.54	7.50	1.47	0.28	9.0401E-10	16.39%
cg09582177	2	21451555		0.15	0.07	0.31	-1.88	0.37	9.628E-10	-9.39%
cg26530319	1	221060923		6.37	3.06	13.24	1.85	0.37	9.7775E-10	9.87%
cg23493016	10	106440778	SORCS3	7.25	3.19	16.48	1.98	0.42	1.0927E-09	10.69%
cg00414835	10	48439452	GDF10	5.88	3.00	11.55	1.77	0.34	1.1096E-09	8.89%
cg08448701	20	21686282	PAX1	5.01	2.66	9.44	1.61	0.32	1.138E-09	15.97%
cg01553433	19	17515486	BST2;MVB12A;BISPR	0.20	0.11	0.36	-1.63	0.31	1.1382E-09	-19.30%
cg02440976	22	22863054	ZNF280B	5.03	2.64	9.62	1.62	0.33	1.1493E-09	9.31%
cg11671688	6	110301075	GPR6	4.75	2.62	8.59	1.56	0.30	1.3377E-09	13.13%
cg01650149	1	240255141	FMN2	4.73	2.60	8.62	1.55	0.31	1.4109E-09	19.84%
cg21294861	14	91125291	TTC7B	0.24	0.15	0.41	-1.41	0.26	1.5548E-09	-17.85%
cg02608002	19	17515778	BST2;MVB12A;BISPR	0.21	0.12	0.38	-1.55	0.30	1.6079E-09	-20.92%
cg19839798	13	108519318	FAM155A	6.90	2.98	15.97	1.93	0.43	1.6423E-09	14.11%
cg02861380	1	75596758	LHX8	6.65	3.05	14.51	1.89	0.40	1.6631E-09	13.12%
cg15443663	16	48936072		4.19	2.46	7.14	1.43	0.27	1.716E-09	15.11%
cg10135483	6	87647527	HTR1E	5.14	2.66	9.93	1.64	0.34	1.9359E-09	16.28%
cg12753587	4	21950584	KCNIP4	4.84	2.62	8.95	1.58	0.31	1.9861E-09	13.85%
cg17233763	8	85095525	RALYL	4.71	2.56	8.66	1.55	0.31	2.2168E-09	18.21%
cg26337020	5	100239022	ST8SIA4	6.49	2.90	14.51	1.87	0.41	2.2266E-09	9.14%
cg18397073	19	42600278	POU2F2	7.40	3.16	17.33	2.00	0.43	2.253E-09	21.14%
cg19098763	1	50513661	ELAVL4	5.23	2.69	10.14	1.65	0.34	2.2809E-09	12.71%

cg02174225	11	133938941	JAM3	6.94	2.87	16.78	1.94	0.45	2.3409E-09	9.80%
cg14100362	5	3326444		4.74	2.59	8.70	1.56	0.31	2.3899E-09	18.54%
cg00849491	18	6510785	LINC01387	0.20	0.11	0.38	-1.60	0.32	2.569E-09	-10.42%
cg27317046	14	74968209	LTBP2	5.18	2.72	9.86	1.64	0.33	2.5719E-09	19.03%
cg25946752	8	50204309		0.22	0.12	0.39	-1.52	0.29	2.7126E-09	-14.40%
cg10530889	10	93805590		5.37	2.77	10.41	1.68	0.34	2.714E-09	11.51%
cg23214755	2	11607042	E2F6	0.22	0.13	0.39	-1.50	0.29	2.7634E-09	-26.60%
cg23322868	9	21993972	CDKN2B-AS1;CDKN2A	0.20	0.11	0.37	-1.59	0.31	2.8197E-09	-19.52%
cg15104158	3	192445388	FGF12	6.73	3.10	14.63	1.91	0.40	3.0139E-09	10.33%
cg00422488	9	100747767	ANP32B	0.22	0.12	0.39	-1.52	0.29	3.1902E-09	-11.04%
cg14745195	2	144011604	ARHGAP15	5.44	2.78	10.63	1.69	0.34	3.3853E-09	16.55%
cg00622837	6	166421992		5.90	2.79	12.47	1.77	0.38	3.4023E-09	11.72%
cg08958294	6	146350131	GRM1	5.57	2.72	11.41	1.72	0.37	3.4412E-09	8.08%
cg26978172	6	31696223	DDAH2	5.86	2.81	12.19	1.77	0.37	4.0337E-09	18.90%
cg05428701	11	129150329		0.23	0.13	0.40	-1.47	0.29	4.0837E-09	-18.24%
cg07624842	X	21958328	SMS	5.32	2.77	10.22	1.67	0.33	4.0871E-09	11.49%
cg01829277	12	101188532	ANO4	5.62	2.74	11.51	1.73	0.37	4.2324E-09	8.75%
cg09297468	6	87647376	HTR1E	6.69	2.98	15.02	1.90	0.41	4.3495E-09	11.81%
cg07709181	8	75545601	MIR2052HG	0.19	0.10	0.37	-1.64	0.33	4.4229E-09	-10.82%
cg05626764	15	26095764	ATP10A	7.62	2.90	19.98	2.03	0.49	4.5449E-09	12.64%
cg13097800	14	47104140		0.15	0.07	0.33	-1.92	0.41	4.5939E-09	-8.87%
cg25175900	12	22854001		4.13	2.41	7.09	1.42	0.28	4.6622E-09	6.16%
cg00000165	1	91194674		6.30	2.93	13.54	1.84	0.39	4.859E-09	11.28%
cg26047066	1	221069136		5.46	2.76	10.77	1.70	0.35	4.9945E-09	24.75%
cg18375860	1	237205409	RYR2	4.48	2.49	8.06	1.50	0.30	5.0034E-09	21.09%
cg04768713	19	53073334	ZNF701	12.76	3.71	43.92	2.55	0.63	5.0314E-09	11.30%
cg14166284	1	221055719	HLX	5.34	2.74	10.40	1.67	0.34	5.0332E-09	15.81%

cg22784954	5	5140646	ADAMTS16	4.15	2.35	7.32	1.42	0.29	5.0813E-09	17.85%
cg11750165	13	112722233	SOX1	5.49	2.68	11.25	1.70	0.37	5.0995E-09	13.11%
cg22288613	4	190962249		5.03	2.62	9.65	1.61	0.33	5.1569E-09	12.00%
cg06873316	11	20690682	NELL1	4.66	2.51	8.65	1.54	0.32	5.2593E-09	17.07%
cg22040301	11	2919798	SLC22A18AS	0.24	0.14	0.42	-1.43	0.28	5.3412E-09	-13.00%
cg12355110	19	57617754		5.40	2.72	10.74	1.69	0.35	5.4488E-09	12.23%
cg07035165	5	113698506	KCNN2	6.30	2.76	14.38	1.84	0.42	5.4841E-09	12.62%
cg07338917	13	112728964		6.00	2.84	12.65	1.79	0.38	5.5517E-09	7.20%
cg04402007	8	35093901	UNC5D	5.66	2.74	11.71	1.73	0.37	5.6008E-09	15.85%
cg14100973	4	87280816	MAPK10	4.80	2.56	9.01	1.57	0.32	5.6872E-09	19.08%
cg04996219	5	11904110	CTNND2	4.81	2.51	9.21	1.57	0.33	5.877E-09	22.39%
cg04597433	4	9783206	DRD5	5.12	2.69	9.74	1.63	0.33	6.025E-09	18.70%
cg01944624	19	58011140	ZNF773	6.76	2.71	16.86	1.91	0.47	6.0726E-09	19.37%
cg14035860	14	55707715		4.88	2.58	9.25	1.59	0.33	6.1374E-09	21.50%
cg21399832	4	117847410		4.77	2.52	9.04	1.56	0.33	6.1638E-09	16.48%
cg15685693	7	70597351	WBSCR17	4.19	2.41	7.29	1.43	0.28	6.2033E-09	18.02%
cg15617814	11	131780492	NTM	4.45	2.44	8.09	1.49	0.31	6.2042E-09	20.74%
cg00977827	1	149192743		5.76	2.64	12.55	1.75	0.40	6.7082E-09	16.23%
cg25001102	1	221068935		5.04	2.59	9.80	1.62	0.34	6.7726E-09	22.19%
cg02661372	1	181056912	IER5	0.26	0.15	0.43	-1.35	0.26	6.7997E-09	-11.13%
cg26755700	20	56689164		4.74	2.54	8.84	1.56	0.32	6.8655E-09	14.57%
cg14099514	5	41510519	PLCXD3	7.17	3.06	16.78	1.97	0.43	6.8752E-09	9.14%
cg08272731	1	75602167	LHX8	4.45	2.47	8.05	1.49	0.30	7.0525E-09	22.53%
cg05615464	15	54270557		8.47	3.31	21.68	2.14	0.48	7.08E-09	9.06%
cg07114310	7	51538921		4.34	2.41	7.82	1.47	0.30	7.1569E-09	11.04%
cg03502002	18	74962133	GALR1	4.96	2.58	9.55	1.60	0.33	7.2511E-09	20.18%
cg25784220	19	58609602	ZSCAN18	4.58	2.45	8.56	1.52	0.32	7.6095E-09	18.40%
cg12810297	1	199717229		4.72	2.52	8.84	1.55	0.32	7.6418E-09	13.22%

cg22001496	8	69243486		4.81	2.49	9.28	1.57	0.34	7.6843E-09	15.96%
cg10071824	13	28366759	GSX1	6.09	2.70	13.75	1.81	0.42	7.8435E-09	16.59%
cg13875518	20	21502218		9.51	3.38	26.73	2.25	0.53	7.9394E-09	9.85%
cg27615388	5	63257092	HTR1A	3.81	2.26	6.41	1.34	0.27	7.9487E-09	12.41%
cg20202112	2	74601685	DCTN1	4.82	2.55	9.11	1.57	0.32	8.0048E-09	15.02%
cg11548303	8	23567350		4.56	2.48	8.37	1.52	0.31	8.0419E-09	13.38%
cg07314384	2	132218291	RNU6-81P	6.15	2.82	13.44	1.82	0.40	8.123E-09	15.61%
cg01142635	4	6224117		4.87	2.55	9.28	1.58	0.33	8.1607E-09	11.59%
cg12354566	15	54270947		10.23	3.58	29.18	2.32	0.53	8.2642E-09	8.53%
cg23207710	3	192126960	FGF12	6.31	2.81	14.16	1.84	0.41	8.2704E-09	12.70%
cg25306915	8	35648588	UNC5D	0.18	0.09	0.37	-1.70	0.35	8.3226E-09	-12.88%
cg26469608	12	113913695		5.73	2.75	11.94	1.75	0.37	8.3329E-09	8.82%
cg08980987	12	25054905	BCAT1	4.78	2.57	8.86	1.56	0.32	8.3331E-09	16.73%
cg01866707	18	45862728		4.12	2.37	7.17	1.42	0.28	8.3441E-09	8.99%
cg06269753	8	72755871	MSC	6.67	2.87	15.53	1.90	0.43	8.3925E-09	12.50%
cg20890325	4	1239370	CTBP1	5.49	2.63	11.46	1.70	0.38	8.6849E-09	18.86%
cg11064255	18	5629890		6.76	2.71	16.82	1.91	0.47	8.8276E-09	15.68%
cg03357798	8	69243285		4.82	2.52	9.24	1.57	0.33	9.1753E-09	12.16%
cg12648074	5	140864474	PCDHGA4	7.33	3.03	17.69	1.99	0.45	9.4402E-09	13.57%
cg02882134	17	46942411	CALCOCO2	3.81	2.25	6.47	1.34	0.27	9.5509E-09	8.91%
cg21678377	2	115919009	DPP10	5.51	2.65	11.44	1.71	0.37	9.8109E-09	16.15%
cg26150462	10	11206868	CUGBP2	7.93	2.99	21.02	2.07	0.50	9.8514E-09	8.43%
cg21331821	19	58545149	ZSCAN1	4.16	2.32	7.48	1.43	0.30	9.9524E-09	9.20%
cg03109827	10	133110349	TCERG1L	4.16	2.37	7.33	1.43	0.29	1.0051E-08	16.17%
cg12664749	1	199717464		4.15	2.41	7.16	1.42	0.28	1.0146E-08	18.24%
cg18716164	19	30019647	VSTM2B	4.43	2.38	8.27	1.49	0.32	1.0157E-08	17.22%
cg00688962	4	21950567	KCNIP4	3.79	2.28	6.28	1.33	0.26	1.0311E-08	20.57%
cg12333537	18	26485722		0.25	0.15	0.43	-1.37	0.27	1.0791E-08	-15.47%

cg23934404	13	112758491		6.31	2.76	14.42	1.84	0.42	1.0839E-08	14.54%
cg11473001	11	131780499	NTM	4.11	2.33	7.23	1.41	0.29	1.0938E-08	15.32%
cg01445580	1	34631891	C1orf94;CSMD2	5.36	2.57	11.17	1.68	0.37	1.1243E-08	15.52%
cg22455450	19	53038972	ZNF808	8.88	3.20	24.63	2.18	0.52	1.127E-08	12.51%
cg15556502	11	43602845	MIR129-2	4.62	2.48	8.62	1.53	0.32	1.1304E-08	15.19%
cg01696784	2	235904501	SH3BP4	4.65	2.48	8.71	1.54	0.32	1.1517E-08	23.89%
cg24211976	15	60285744		4.81	2.59	8.94	1.57	0.32	1.1973E-08	29.64%
cg23591955	14	52294766		0.25	0.14	0.42	-1.40	0.28	1.2029E-08	-10.66%
cg16389285	15	26108399	ATP10A	4.40	2.36	8.23	1.48	0.32	1.21E-08	15.11%
cg12492087	15	42749885	ZFP106	4.34	2.41	7.80	1.47	0.30	1.221E-08	22.84%
cg26183190	6	33754751	LEMD2	4.12	2.30	7.37	1.42	0.30	1.2224E-08	10.34%
cg08396985	2	5896489		4.56	2.47	8.42	1.52	0.31	1.2305E-08	27.72%
cg04711162	19	30017511	VSTM2B	5.98	2.69	13.29	1.79	0.41	1.2519E-08	13.82%
cg06792417	3	137487857		7.04	2.96	16.76	1.95	0.44	1.2732E-08	9.72%
cg22446630	9	126360170	DENND1A	3.86	2.25	6.61	1.35	0.27	1.2909E-08	11.65%
cg23843400	11	10747348		0.26	0.15	0.44	-1.35	0.28	1.3119E-08	-5.42%
cg00116234	9	18474243	ADAMTSL1	6.53	2.84	15.01	1.88	0.42	1.3129E-08	11.43%
cg16927040	3	187388128	SST	5.33	2.62	10.84	1.67	0.36	1.3174E-08	14.12%
cg25514475	5	171693762	UBTD2	4.16	2.39	7.25	1.43	0.28	1.3221E-08	16.69%
cg08194879	6	123317569	CLVS2	3.73	2.21	6.29	1.32	0.27	1.3421E-08	13.87%
cg01972979	X	133680446	MGC16121;MIR503	4.53	2.47	8.31	1.51	0.31	1.3651E-08	20.11%
cg24426044	3	178254281	KCNMB2;KCNMB2-AS1	4.57	2.47	8.47	1.52	0.31	1.3818E-08	11.83%
cg03323696	5	59189120	PDE4D	4.64	2.51	8.60	1.53	0.31	1.3825E-08	12.54%
cg09868598	5	50674015		4.95	2.56	9.56	1.60	0.34	1.3959E-08	11.70%
cg25407557	12	132168981		3.90	2.29	6.67	1.36	0.27	1.4125E-08	6.90%
cg15160644	6	151335863	MTHFD1L	3.31	2.08	5.27	1.20	0.24	1.4373E-08	4.38%
cg16415863	19	17516339	BST2;MVB12A;BISPR	0.25	0.14	0.43	-1.40	0.28	1.4438E-08	-17.58%

cg00266009	8	85095275	RALYL	8.89	3.18	24.89	2.18	0.53	1.4532E-08	8.02%
cg04819716	7	153749318	DPP6	5.06	2.57	9.99	1.62	0.35	1.492E-08	16.35%
cg22376706	12	71314612	PTPRR	5.06	2.54	10.09	1.62	0.35	1.516E-08	12.76%
cg16587707	11	2920265	SLC22A18AS	0.20	0.11	0.39	-1.59	0.33	1.5325E-08	-8.78%
cg09040797	12	63544768	AVPR1A	4.54	2.44	8.46	1.51	0.32	1.5411E-08	17.97%
cg02292901	10	11001688		0.20	0.10	0.39	-1.62	0.35	1.5552E-08	-10.06%
cg19469068	8	69243293	C8orf34;C8orf34-AS1	3.79	2.22	6.49	1.33	0.27	1.5586E-08	19.37%
cg06051311	6	30131001	TRIM15	0.28	0.17	0.47	-1.26	0.26	1.6125E-08	-12.17%
cg26691307	5	113698769	KCNN2	7.25	3.13	16.79	1.98	0.43	1.6163E-08	7.61%
cg07488625	19	13617171	CACNA1A	4.61	2.35	9.05	1.53	0.34	1.6478E-08	13.28%
cg16393481	7	155607461		0.27	0.17	0.45	-1.29	0.25	1.6522E-08	-9.47%
cg06820905	8	69243752		5.53	2.56	11.94	1.71	0.39	1.6834E-08	11.39%
cg22370295	1	237205384	RYR2	4.41	2.43	7.99	1.48	0.30	1.6892E-08	20.80%
cg07369950	19	58609987	ZSCAN18	6.72	2.81	16.06	1.91	0.44	1.6997E-08	10.80%
cg14367229	13	28367741	GSX1	6.69	2.85	15.70	1.90	0.44	1.7019E-08	10.70%
cg05827312	13	28394966		5.42	2.66	11.03	1.69	0.36	1.7191E-08	10.36%
cg10124812	2	54087517	GPR75;LOC100302652	4.35	2.42	7.81	1.47	0.30	1.732E-08	13.50%
cg14060496	20	61638518	BHLHE23	4.07	2.28	7.25	1.40	0.29	1.7549E-08	17.25%
cg04436994	1	221055665	HLX	4.30	2.40	7.70	1.46	0.30	1.7654E-08	15.35%
cg20082563	5	10485736		0.25	0.15	0.43	-1.38	0.28	1.7954E-08	-17.76%
cg02721902	17	46825371		4.61	2.44	8.67	1.53	0.32	1.8169E-08	12.23%
cg09977449	17	50236098	CA10	5.00	2.54	9.84	1.61	0.35	1.8484E-08	15.45%
cg23688510	6	166581929	T	10.69	3.05	37.51	2.37	0.64	1.9116E-08	13.26%
cg08146483	1	75602412	LHX8	4.17	2.33	7.46	1.43	0.30	1.914E-08	14.81%
cg15669183	13	108518621	FAM155A	5.57	2.64	11.73	1.72	0.38	1.938E-08	12.08%
cg18881778	12	120241287	CIT	4.01	2.31	6.98	1.39	0.28	1.9493E-08	15.44%
cg20311863	1	91184126	BARHL2	4.93	2.48	9.80	1.60	0.35	1.959E-08	16.46%

cg18780257	3	192126023	FGF12	4.89	2.50	9.58	1.59	0.34	1.9885E-08	17.57%
cg27607773	2	28066049	RBKS	4.32	2.42	7.72	1.46	0.30	1.9909E-08	21.52%
cg22579471	5	55199404	IL31RA	0.26	0.15	0.44	-1.34	0.27	2.0031E-08	-11.13%
cg21236576	15	28371423	HERC2	3.86	2.24	6.64	1.35	0.28	2.0465E-08	10.96%
cg25095032	6	31696423	DDAH2	5.36	2.63	10.90	1.68	0.36	2.0617E-08	21.16%
cg07129725	19	56915266	ZNF583	4.99	2.44	10.18	1.61	0.36	2.0798E-08	7.46%
cg25570291	1	158985360	IFI16	0.27	0.16	0.45	-1.32	0.27	2.0935E-08	-14.22%
cg23615676	5	113697632	KCNN2	7.36	2.94	18.46	2.00	0.47	2.1028E-08	9.15%
cg12055940	11	71421279		4.08	2.32	7.19	1.41	0.29	2.1348E-08	5.71%
cg17165848	2	175462452	WIPF1	0.25	0.14	0.43	-1.40	0.28	2.1833E-08	-10.39%
cg07557260	8	132052870	ADCY8	4.77	2.47	9.21	1.56	0.34	2.2155E-08	12.37%
cg00757182	19	58011393	ZNF773	4.73	2.38	9.40	1.55	0.35	2.2294E-08	12.17%
cg13471424	1	149223500		5.54	2.63	11.70	1.71	0.38	2.2456E-08	13.08%
cg19717586	11	131781257	NTM	4.33	2.35	7.95	1.46	0.31	2.2561E-08	25.33%
cg23642130	19	30866365	ZNF536	4.26	2.35	7.72	1.45	0.30	2.2644E-08	18.13%
cg12785694	3	160122168	SMC4;MIR15B;MIR16-2	0.24	0.14	0.43	-1.42	0.29	2.2794E-08	-20.56%
cg16023896	11	3250827	MRGPRE	0.16	0.07	0.35	-1.83	0.40	2.2811E-08	-7.38%
cg18191540	14	107281437		0.26	0.15	0.43	-1.36	0.27	2.2818E-08	-16.85%
cg20407119	11	56549867		0.24	0.14	0.42	-1.42	0.29	2.3048E-08	-9.71%
cg17393267	3	192127356	FGF12	8.74	2.72	28.08	2.17	0.60	2.3089E-08	13.21%
cg03881775	3	147111135	ZIC4	4.17	2.31	7.51	1.43	0.30	2.3182E-08	14.94%
cg10982443	14	20903611	KLHL33	5.04	2.54	9.99	1.62	0.35	2.3321E-08	23.49%
cg16055378	19	30015991	VSTM2B;LOC284395	4.05	2.29	7.17	1.40	0.29	2.3568E-08	15.70%
cg09876519	6	50675610		6.22	2.82	13.73	1.83	0.40	2.3591E-08	10.02%
cg12613383	1	242688681	PLD5	6.87	2.91	16.23	1.93	0.44	2.36E-08	8.96%
cg00688840	14	106363051		0.27	0.16	0.45	-1.32	0.27	2.3836E-08	-9.83%
cg06600429	5	160975956	GABRB2	6.75	2.89	15.79	1.91	0.43	2.398E-08	12.22%

cg05468843	11	117857990	IL10RA	0.26	0.15	0.45	-1.33	0.28	2.4006E-08	-11.26%
cg17535595	13	53422808	PCDH8	4.81	2.41	9.62	1.57	0.35	2.4113E-08	10.73%
cg08572394	5	76932996	OTP	5.37	2.60	11.11	1.68	0.37	2.4355E-08	14.30%
cg19723208	1	62996435	DOCK7	4.41	2.40	8.10	1.48	0.31	2.4367E-08	17.65%
cg04580344	6	127797022	C6orf174	3.81	2.21	6.58	1.34	0.28	2.4637E-08	7.38%
cg12553083	1	108492681	VAV3	0.26	0.15	0.45	-1.34	0.27	2.4793E-08	-15.73%
cg18115064	14	48143618	MDGA2	8.86	3.16	24.84	2.18	0.53	2.5082E-08	9.43%
cg10237903	8	139509075	FAM135B	3.84	2.22	6.63	1.34	0.28	2.5326E-08	19.70%
cg22489957	6	28921805		4.69	2.48	8.90	1.55	0.33	2.548E-08	9.78%
cg11877129	12	128752356	TMEM132C	6.61	2.83	15.44	1.89	0.43	2.5483E-08	10.19%
cg10778841	10	106401319	SORCS3	3.65	2.18	6.11	1.30	0.26	2.5714E-08	18.10%
cg22049569	19	54466538	CACNG8	4.17	2.30	7.54	1.43	0.30	2.6019E-08	10.13%
cg08790440	2	124782831	CNTNAP5	4.02	2.26	7.13	1.39	0.29	2.6052E-08	17.99%
cg09989037	1	44300942	ST3GAL3	0.29	0.18	0.47	-1.25	0.25	2.6088E-08	-10.64%
cg13849378	1	241520331	RGS7	4.08	2.26	7.38	1.41	0.30	2.6229E-08	21.03%
cg19928294	6	102943909		0.26	0.15	0.44	-1.37	0.28	2.639E-08	-10.73%
cg12746465	20	57782636	ZNF831	0.20	0.10	0.39	-1.60	0.33	2.655E-08	-9.19%
cg09170366	3	152374157		4.65	2.38	9.08	1.54	0.34	2.6615E-08	12.05%
cg12431123	12	103351432	ASCL1	7.05	2.87	17.33	1.95	0.46	2.6913E-08	13.94%
cg10182317	6	123317487	CLVS2	3.96	2.28	6.88	1.38	0.28	2.6919E-08	18.72%
cg23861668	11	20691126	NELL1	4.46	2.38	8.34	1.50	0.32	2.6957E-08	18.22%
cg07726288	1	248855278		3.87	2.24	6.68	1.35	0.28	2.7063E-08	7.48%
cg22457238	19	58570995	ZNF135	5.77	2.62	12.68	1.75	0.40	2.7075E-08	11.50%
cg20318143	19	17516370	BST2;MVB12A;BISPR	0.26	0.15	0.44	-1.37	0.28	2.716E-08	-12.64%
cg22038124	15	51396175	TNFAIP8L3	4.15	2.32	7.42	1.42	0.30	2.7179E-08	14.60%
cg03732161	9	18473918	ADAMTSL1	4.70	2.44	9.05	1.55	0.33	2.7309E-08	12.52%
cg24238849	20	21686715	PAX1	5.80	2.68	12.51	1.76	0.39	2.7361E-08	9.97%

cg18729357	19	53496893	ZNF702P	5.12	2.53	10.35	1.63	0.36	2.7515E-08	11.86%
cg23034757	5	140787864	PCDHGA4	6.08	2.75	13.47	1.81	0.41	2.7726E-08	11.10%
cg14519115	5	41510650	PLCXD3	8.20	3.11	21.62	2.10	0.49	2.7879E-08	8.78%
cg08326559	7	103498178	RELN	3.97	2.25	6.99	1.38	0.29	2.8243E-08	12.53%
cg06735418	6	55911430		0.29	0.18	0.48	-1.23	0.25	2.8331E-08	-18.57%
cg10691901	9	74062074		5.49	2.60	11.60	1.70	0.38	2.883E-08	11.26%
cg15638709	10	110226387		4.08	2.30	7.22	1.41	0.29	2.9122E-08	18.95%
cg13445796	11	20181725	DBX1	4.41	2.35	8.26	1.48	0.32	2.933E-08	15.30%
cg12169233	19	11959578		8.79	2.81	27.49	2.17	0.58	2.9426E-08	11.94%
cg03192421	20	10643245	JAG1	3.94	2.25	6.90	1.37	0.29	2.9497E-08	17.01%
cg14323415	X	7909363		3.80	2.21	6.54	1.34	0.28	2.9564E-08	9.03%
cg09632273	6	31696229	DDAH2	4.45	2.40	8.25	1.49	0.31	2.9663E-08	21.21%
cg25282723	2	208174949		3.72	2.23	6.21	1.31	0.26	2.9909E-08	15.26%
cg10769146	1	214156644		6.75	2.86	15.95	1.91	0.44	2.9982E-08	6.43%
cg21093192	19	54412993		5.58	2.58	12.07	1.72	0.39	3.0107E-08	14.70%
cg05353133	1	229567914	ACTA1	4.78	2.46	9.29	1.57	0.34	3.0304E-08	9.09%
cg03313364	17	40440904	STAT5A	0.23	0.13	0.43	-1.45	0.31	3.036E-08	-10.84%
cg00038681	3	77517922	ROBO2	4.11	2.33	7.24	1.41	0.29	3.0644E-08	15.88%
cg19632836	13	112332213		4.01	2.28	7.06	1.39	0.29	3.0648E-08	20.07%
cg26549174	19	10381355	ICAM1	0.26	0.15	0.44	-1.35	0.27	3.0855E-08	-11.57%
cg02763101	19	56904945	ZNF582	5.15	2.41	10.97	1.64	0.39	3.0896E-08	21.50%
cg23095192	2	145271307	ZEB2	4.50	2.41	8.41	1.50	0.32	3.1146E-08	11.92%
cg27273868	6	162075441	PARK2	0.26	0.15	0.45	-1.35	0.28	3.1178E-08	-4.64%
cg22702328	17	50235965	CA10	4.76	2.45	9.25	1.56	0.34	3.1385E-08	15.64%
cg03684768	10	120000694		4.08	2.25	7.41	1.41	0.30	3.1472E-08	16.16%
cg23108778	1	7616070	CAMTA1	0.26	0.16	0.45	-1.33	0.27	3.1542E-08	-11.76%
cg05336115	20	983104	RSPO4	6.25	2.71	14.41	1.83	0.43	3.1665E-08	11.14%
cg16988262	1	15930761		0.27	0.15	0.46	-1.33	0.29	3.1833E-08	-14.64%

cg11338643	6	166580983	T	6.94	2.83	17.02	1.94	0.46	3.1908E-08	11.97%
cg12006284	11	32449638	WT1	4.25	2.34	7.70	1.45	0.30	3.2022E-08	11.44%
cg14972743	19	58609960	ZSCAN18	5.36	2.57	11.16	1.68	0.37	3.2058E-08	12.31%
cg11850773	18	904963	ADCYAP1	4.17	2.32	7.51	1.43	0.30	3.2094E-08	15.41%
cg10494860	20	62693951	TCEA2	4.20	2.36	7.48	1.44	0.29	3.2573E-08	14.53%
cg16451391	5	70741802		4.26	2.37	7.67	1.45	0.30	3.2634E-08	10.51%
cg07194250	X	133680463	MGC16121;MIR503	4.35	2.39	7.90	1.47	0.31	3.2971E-08	14.09%
cg05057720	14	38724675	CLEC14A	3.78	2.21	6.47	1.33	0.27	3.3084E-08	18.84%
cg24348495	20	62693971	TCEA2	4.58	2.45	8.59	1.52	0.32	3.3156E-08	21.23%
cg14323675	9	17907018		6.00	2.72	13.21	1.79	0.40	3.3246E-08	11.95%
cg21497056	7	70597354	WBSCR17	3.80	2.23	6.47	1.33	0.27	3.3429E-08	17.97%
cg07393736	3	172167810		5.59	2.64	11.83	1.72	0.38	3.3453E-08	14.05%
cg10676084	15	27017560	GABRB3	4.85	2.46	9.54	1.58	0.35	3.3525E-08	11.36%
cg16509658	5	160975347	GABRB2	4.63	2.29	9.35	1.53	0.36	3.359E-08	10.15%
cg22762091	8	132052843	ADCY8	3.97	2.26	6.97	1.38	0.29	3.3718E-08	16.63%
cg03307893	15	26108683	ATP10A	4.99	2.39	10.43	1.61	0.38	3.3889E-08	12.50%
cg20167366	6	30094980		4.77	2.44	9.34	1.56	0.34	3.4363E-08	13.97%
cg05181865	19	58570652	ZNF135	3.55	2.11	5.95	1.27	0.26	3.4392E-08	10.12%
cg04475027	12	128752040	TMEM132C	4.74	2.44	9.22	1.56	0.34	3.4462E-08	16.59%
cg08074851	2	47797590	KCNK12	5.03	2.46	10.25	1.61	0.36	3.4747E-08	14.99%
cg18072285	5	95808995	LOC101929710	0.28	0.17	0.46	-1.28	0.26	3.5514E-08	-10.23%
cg24621437	10	108924560	SORCS1	4.40	2.34	8.26	1.48	0.32	3.5597E-08	14.78%
cg03818930	6	39095898		0.26	0.16	0.45	-1.33	0.27	3.6059E-08	-8.34%
cg04513294	6	40919057	LOC101929555	3.73	2.17	6.42	1.32	0.28	3.6438E-08	14.04%
cg13999330	7	2472814	CHST12	6.08	2.62	14.06	1.80	0.43	3.6495E-08	12.10%
cg06330323	16	2132315	TSC2	4.95	2.46	9.96	1.60	0.36	3.6703E-08	23.04%
cg19058262	19	1231292	C19orf26	4.06	2.25	7.36	1.40	0.30	3.6723E-08	8.95%
cg01395723	5	113696634	KCNN2	5.07	2.46	10.43	1.62	0.37	3.6779E-08	14.28%

cg26245879	3	10375226	ATP2B2	0.27	0.16	0.45	-1.32	0.26	3.7217E-08	-7.56%
cg22976218	2	182544321	NEUROD1	6.16	2.78	13.62	1.82	0.41	3.7313E-08	11.39%
cg08851784	5	22212938	CDH12	4.32	2.36	7.90	1.46	0.31	3.747E-08	10.21%
cg07733830	7	63768483		3.88	2.24	6.70	1.35	0.28	3.7474E-08	14.87%
cg22047387	12	55413872	NEUROD4	5.42	2.58	11.40	1.69	0.38	3.7518E-08	10.55%
cg04471914	7	153749310	DPP6	4.08	2.29	7.29	1.41	0.30	3.7554E-08	18.02%
cg01718742	16	62070184	CDH8	4.09	2.30	7.25	1.41	0.29	3.7657E-08	13.60%
cg10119075	10	7709738	ITIH5	6.10	2.67	13.93	1.81	0.42	3.7847E-08	11.77%
cg14724613	7	86273429	GRM3	7.66	3.00	19.54	2.04	0.48	3.7941E-08	9.28%
cg14403099	X	51360708		3.61	2.15	6.07	1.28	0.26	3.8046E-08	7.34%
cg09840146	5	140705747		5.65	2.62	12.17	1.73	0.39	3.8081E-08	11.31%
cg13135595	18	77558846		4.88	2.35	10.14	1.59	0.37	3.8828E-08	10.43%
cg11227141	1	2003650	PRKCZ	3.36	2.06	5.49	1.21	0.25	3.8846E-08	8.79%
cg04094811	6	1393336	FOXF2	6.78	2.80	16.38	1.91	0.45	3.8896E-08	12.53%
cg00816164	3	20394477		0.24	0.13	0.43	-1.42	0.30	3.917E-08	-13.87%
cg14119999	13	108518445	FAM155A	6.37	2.72	14.88	1.85	0.43	3.9256E-08	12.52%
cg09577391	19	58515046	ZNF606	4.13	2.31	7.36	1.42	0.30	3.9361E-08	9.23%
cg16638540	19	58570468	ZNF135	4.93	2.49	9.77	1.59	0.35	3.9487E-08	12.32%
cg14445507	7	152890360		4.26	2.28	7.94	1.45	0.32	3.9489E-08	8.82%
cg19839825	19	7616011	PNPLA6	4.64	2.44	8.83	1.54	0.33	3.9505E-08	17.34%
cg11224418	21	35897663	RCAN1	3.92	2.25	6.80	1.37	0.28	3.9651E-08	19.35%
cg01485075	19	22817371	ZNF492	4.84	2.38	9.83	1.58	0.36	3.9666E-08	14.69%
cg06640533	16	58197401	CSNK2A2	3.18	2.00	5.05	1.16	0.24	3.9995E-08	4.86%
cg18210226	6	123317714	CLVS2	4.40	2.37	8.17	1.48	0.32	4.0132E-08	15.29%
cg26609631	13	28366814	GSX1	3.79	2.19	6.56	1.33	0.28	4.0183E-08	21.51%
cg22607266	1	199717290		3.83	2.24	6.55	1.34	0.27	4.032E-08	17.18%
cg16661508	12	79258427	SYT1	5.00	2.48	10.10	1.61	0.36	4.0362E-08	12.37%
cg09406615	3	165555103	BCHE	4.45	2.36	8.40	1.49	0.32	4.0585E-08	14.20%

cg23245720	X	133680549	MGC16121;MIR503	4.52	2.38	8.58	1.51	0.33	4.0615E-08	15.72%
cg03189837	5	59817962	PART1	0.27	0.16	0.46	-1.30	0.26	4.079E-08	-10.71%
cg00446413	7	153749206	DPP6	4.81	2.42	9.56	1.57	0.35	4.1447E-08	13.29%
cg07166360	3	137483071	SOX14	4.90	2.41	9.96	1.59	0.36	4.1468E-08	10.47%
cg11098290	17	63355141		0.21	0.11	0.41	-1.57	0.34	4.1505E-08	-6.76%
cg15148973	10	122333392	PPAPDC1A	0.25	0.14	0.44	-1.38	0.29	4.1509E-08	-12.35%
cg12641543	19	57615001		5.49	2.58	11.67	1.70	0.39	4.1563E-08	10.30%
cg04023663	7	7031200		3.54	2.14	5.86	1.26	0.26	4.1826E-08	9.31%
cg20450979	4	176923542	GPM6A	5.23	2.41	11.35	1.65	0.40	4.2242E-08	11.37%
cg01041222	4	158142863	GRIA2	5.21	2.56	10.62	1.65	0.36	4.2299E-08	7.54%
cg25830612	22	26242380	MYO18B	0.26	0.16	0.45	-1.33	0.27	4.2483E-08	-12.21%
cg12976081	3	147105899	ZIC4	6.16	2.71	14.00	1.82	0.42	4.2506E-08	10.03%
cg11465936	X	152647550		0.27	0.16	0.46	-1.30	0.26	4.3047E-08	-5.91%
cg19844799	20	39439001		0.22	0.12	0.41	-1.51	0.32	4.3171E-08	-8.12%
cg05923226	19	15121516	CCDC105	5.03	2.53	10.01	1.62	0.35	4.333E-08	9.23%
cg04467624	20	55799928	BMP7	3.84	2.23	6.60	1.35	0.28	4.359E-08	13.99%
cg23055735	11	70557464	SHANK2	3.94	2.27	6.85	1.37	0.28	4.4071E-08	14.90%
cg11595545	1	111217497	KCNA3	5.19	2.41	11.19	1.65	0.39	4.4293E-08	19.45%
cg22164298	4	114276834	ANK2	0.29	0.18	0.47	-1.25	0.25	4.4821E-08	-15.25%
cg00800512	19	58095518	ZIK1	4.72	2.37	9.40	1.55	0.35	4.5252E-08	13.38%
cg21660452	11	64397807	NRXN2	5.19	2.62	10.30	1.65	0.35	4.5984E-08	20.48%
cg22685409	11	30038692	KCNA4	4.98	2.46	10.10	1.61	0.36	4.6135E-08	10.60%
cg10746084	6	169654126	THBS2	4.19	2.30	7.63	1.43	0.31	4.6272E-08	6.61%
cg08620154	8	69244553		6.88	2.64	17.89	1.93	0.49	4.6299E-08	13.39%
cg27528660	8	72987924	TRPA1	4.48	2.37	8.46	1.50	0.32	4.6313E-08	13.97%
cg15489250	14	36994906		6.36	2.57	15.74	1.85	0.46	4.6451E-08	14.82%
cg11014373	2	105479054		5.08	2.48	10.40	1.62	0.37	4.6547E-08	13.70%
cg23878255	7	141645174	CLEC5A	0.27	0.16	0.46	-1.31	0.27	4.6607E-08	-20.64%

cg23664334	1	149288143	LOC388692	4.78	2.45	9.36	1.57	0.34	4.7172E-08	6.26%
cg02139965	5	169739749		0.27	0.16	0.46	-1.30	0.27	4.7362E-08	-14.84%
cg22478591	4	176923544	GPM6A	6.53	2.67	15.97	1.88	0.46	4.7363E-08	9.92%
cg23673107	19	11908712	ZNF491	4.05	2.25	7.29	1.40	0.30	4.7385E-08	7.23%
cg21130374	21	42734266	MX2	0.26	0.15	0.45	-1.33	0.27	4.7514E-08	-20.94%
cg09111484	12	80749651		0.24	0.13	0.44	-1.43	0.31	4.78E-08	-5.86%
cg14043737	20	61637672	BHLHE23	5.14	2.50	10.56	1.64	0.37	4.8021E-08	9.66%
cg25734490	12	103351188	ASCL1	5.55	2.49	12.34	1.71	0.41	4.8209E-08	8.10%
cg24520975	6	31651362		3.80	2.21	6.52	1.34	0.28	4.843E-08	13.61%
cg19822343	5	180527495		6.10	2.67	13.92	1.81	0.42	4.8738E-08	10.59%
cg14069287	4	169631613	PALLD	4.25	2.36	7.66	1.45	0.30	4.8776E-08	10.44%
cg26490272	8	72756153	MSC;MSC-AS1	4.11	2.22	7.62	1.41	0.31	4.8874E-08	19.56%
cg14023149	17	72199483	RPL38	6.05	2.72	13.43	1.80	0.41	4.9039E-08	9.08%
cg03239178	12	103358881		6.27	2.70	14.54	1.84	0.43	4.9293E-08	10.34%
cg10866298	8	9760869	LOC157627	14.33	3.71	55.27	2.66	0.69	4.9475E-08	11.03%
cg07537187	9	88669060	GOLM1	3.34	2.05	5.43	1.21	0.25	4.9541E-08	8.10%
cg12373617	5	113698140	KCNN2	7.23	2.65	19.69	1.98	0.51	4.9729E-08	16.95%
cg03075534	1	240161447		3.34	2.07	5.40	1.21	0.24	4.9775E-08	17.96%
cg16430072	5	113696863	KCNN2	15.62	3.37	72.47	2.75	0.78	4.9976E-08	11.54%
cg13209481	14	36973483	SFTA3	4.57	2.35	8.88	1.52	0.34	5.039E-08	15.46%
cg06801852	14	105622992	JAG2	3.67	2.17	6.19	1.30	0.27	5.0757E-08	11.54%
cg19010490	15	37388127	MEIS2	4.04	2.23	7.34	1.40	0.30	5.0925E-08	16.60%
cg08481464	10	77871618	C10orf11	3.97	2.26	6.98	1.38	0.29	5.1114E-08	14.34%
cg15499265	9	117157330	AKNA	3.81	2.23	6.52	1.34	0.27	5.1401E-08	9.95%
cg20405017	17	50235393	CA10	3.92	2.24	6.89	1.37	0.29	5.188E-08	19.45%
cg24124798	19	58400275	ZNF814	5.41	2.52	11.59	1.69	0.39	5.2512E-08	9.45%
cg16020747	8	85095394	RALYL	3.67	2.17	6.21	1.30	0.27	5.2602E-08	26.21%
cg26848718	11	32454975	WT1	5.03	2.49	10.16	1.62	0.36	5.2756E-08	7.17%

cg07362849	19	17516901	BISPR;BST2;MVB12A	0.27	0.16	0.46	-1.30	0.27	5.2825E-08	-11.91%
cg07837260	9	19788564		5.31	2.57	10.97	1.67	0.37	5.2849E-08	10.91%
cg08812936	3	178254213	KCNMB2	3.97	2.24	7.05	1.38	0.29	5.2893E-08	11.94%
cg11687330	11	132953239	OPCML	5.31	2.47	11.38	1.67	0.39	5.3558E-08	12.80%
cg15498379	19	58609981	ZSCAN18	5.58	2.55	12.23	1.72	0.40	5.3681E-08	13.12%
cg13065624	1	170253617	LOC284688	3.59	2.15	5.98	1.28	0.26	5.3799E-08	14.47%
cg16304950	18	5543945	EPB41L3	6.79	2.66	17.36	1.92	0.48	5.3971E-08	13.79%
cg13155726	20	55800007	BMP7	3.88	2.22	6.78	1.36	0.28	5.4024E-08	8.68%
cg04034967	7	1309108		3.80	2.19	6.60	1.34	0.28	5.4148E-08	18.08%
cg19590598	2	127782813		4.14	2.29	7.51	1.42	0.30	5.4365E-08	12.54%
cg10706013	21	44104950	PDE9A	0.27	0.16	0.46	-1.31	0.27	5.4429E-08	-16.34%
cg13239420	1	240161249		4.10	2.26	7.45	1.41	0.30	5.4558E-08	17.81%
cg08061720	16	77619380		0.26	0.15	0.45	-1.36	0.28	5.4761E-08	-13.95%
cg18063733	5	50265443		4.50	2.34	8.65	1.50	0.33	5.5228E-08	13.76%
cg16862704	6	155189348		3.82	2.13	6.88	1.34	0.30	5.6329E-08	16.80%
cg09515921	1	221057558	HLX	4.33	2.36	7.96	1.47	0.31	5.6334E-08	22.63%
cg20229496	19	35395800		4.34	2.33	8.05	1.47	0.32	5.6397E-08	8.44%
cg26155697	5	52150386	ITGA1	4.00	2.26	7.08	1.39	0.29	5.6551E-08	15.90%
cg21806580	5	18746010		0.24	0.14	0.43	-1.42	0.30	5.6701E-08	-13.19%
cg15665203	13	112282481		3.76	2.21	6.40	1.32	0.27	5.6735E-08	16.17%
cg01874697	10	106401517	SORCS3	4.94	2.44	10.00	1.60	0.36	5.7161E-08	13.41%
cg03701266	5	10649678	ANKRD33B	4.12	2.31	7.36	1.42	0.30	5.7261E-08	9.21%
cg25114913	6	96463959	FUT9	4.67	2.37	9.23	1.54	0.35	5.7465E-08	14.38%
cg18702971	22	29920546	THOC5	3.48	2.08	5.82	1.25	0.26	5.7477E-08	6.37%
cg19484548	14	100953108	WDR25	3.59	2.11	6.11	1.28	0.27	5.7826E-08	12.26%
cg11325267	3	137480853		4.43	2.33	8.43	1.49	0.33	5.7868E-08	10.27%
cg22269543	1	214156497	PROX1-AS1	6.14	2.57	14.69	1.82	0.45	5.8661E-08	9.81%

cg03045635	4	9783198	DRD5		3.95	2.25	6.91	1.37	0.29	5.8747E-08	17.47%

Supplementary Table 2. Prediction performance as measured by the sensitivity and specificity for logistic models including cg16767801 and/or cg23642047 as predictor(s) and comparing controls (N=114) to all cases (CIN3 and Cancer) (A, N=127), CIN3 cases only (B, N=73) or cancer cases only (C, N=54). Performance metrics are reported as the mean, 5th and 95th quantiles computed over N=1,000 subsamples for different proportions of the population predicted as cases.

A

	cg16767801			cg23642047			cg16767801 + cg23642047	
Proportion	Sensitivity	Specificity	Proportion	Sensitivity	Specificity	Proportion	Sensitivity	Specificity
10%	0.97 [0.96-0.97]	0.17 [0.17-0.18]	10%	0.95 [0.94-0.96]	0.15 [0.14-0.17]	10%	1.00 [0.99-1.00]	0.21 [0.20-0.21]
20%	0.91 [0.91-0.92]	0.32 [0.32-0.33]	20%	0.91 [0.90-0.91]	0.32 [0.31-0.32]	20%	0.96 [0.95-0.98]	0.38 [0.37-0.39]
30%	0.86 [0.85-0.87]	0.48 [0.46-0.49]	30%	0.85 [0.84-0.86]	0.47 [0.46-0.47]	30%	0.93 [0.93-0.94]	0.56 [0.55-0.57]
40%	0.83 [0.82-0.83]	0.65 [0.64-0.65]	40%	0.75 [0.74-0.76]	0.56 [0.55-0.57]	40%	0.88 [0.87-0.89]	0.71 [0.70-0.72]
50%	0.77 [0.76-0.78]	0.81 [0.80-0.82]	50%	0.68 [0.68-0.69]	0.71 [0.70-0.71]	50%	0.83 [0.83-0.83]	0.87 [0.87-0.88]
60%	0.70 [0.69-0.70]	0.94 [0.93-0.94]	60%	0.56 [0.55-0.57]	0.78 [0.77-0.79]	60%	0.72 [0.71-0.72]	0.95 [0.95-0.96]
70%	0.56 [0.56-0.56]	0.99 [0.99-0.99]	70%	0.46 [0.45-0.46]	0.88 [0.87-0.89]	70%	0.56 [0.56-0.57]	1.00 [0.99-1.00]
80%	0.38 [0.37-0.38]	1.00 [0.99-1.00]	80%	0.33 [0.32-0.33]	0.94 [0.94-0.95]	80%	0.38 [0.38-0.38]	1.00 [1.00-1.00]
90%	0.19 [0.19-0.19]	1.00 [1.00-1.00]	90%	0.19 [0.19-0.19]	1.00 [1.00-1.00]	90%	0.19 [0.19-0.19]	1.00 [1.00-1.00]

B

	cg16767801			cg23642047			cg16767801 + cg23642047	
Proportion	Sensitivity	Specificity	Proportion	Sensitivity	Specificity	Proportion	Sensitivity	Specificity
10%	0.95 [0.95-0.96]	0.13 [0.12-0.13]	10%	0.95 [0.93-0.97]	0.13 [0.11-0.14]	10%	1.00 [1.00-1.00]	0.16 [0.16-0.16]
20%	0.90 [0.88-0.90]	0.26 [0.25-0.26]	20%	0.91 [0.90-0.93]	0.27 [0.26-0.28]	20%	0.97 [0.95-0.97]	0.30 [0.29-0.31]
30%	0.85 [0.84-0.86]	0.40 [0.39-0.40]	30%	0.87 [0.85-0.88]	0.41 [0.39-0.41]	30%	0.93 [0.93-0.95]	0.45 [0.45-0.46]
40%	0.80 [0.78-0.82]	0.53 [0.52-0.54]	40%	0.78 [0.77-0.79]	0.52 [0.51-0.53]	40%	0.90 [0.89-0.90]	0.59 [0.59-0.60]
50%	0.76 [0.74-0.77]	0.67 [0.66-0.68]	50%	0.70 [0.70-0.70]	0.63 [0.63-0.63]	50%	0.83 [0.82-0.84]	0.71 [0.71-0.72]
60%	0.72 [0.70-0.73]	0.80 [0.79-0.81]	60%	0.62 [0.62-0.63]	0.74 [0.74-0.75]	60%	0.74 [0.73-0.75]	0.82 [0.81-0.82]
70%	0.62 [0.62-0.63]	0.91 [0.90-0.91]	70%	0.47 [0.45-0.48]	0.81 [0.80-0.82]	70%	0.67 [0.66-0.68]	0.94 [0.93-0.95]
80%	0.49 [0.49-0.49]	0.99 [0.99-0.99]	80%	0.36 [0.34-0.37]	0.91 [0.89-0.91]	80%	0.48 [0.47-0.48]	0.98 [0.97-0.98]
90%	0.23 [0.23-0.23]	0.99 [0.99-0.99]	90%	0.20 [0.18-0.22]	0.97 [0.96-0.98]	90%	0.25 [0.25-0.25]	1.00 [1.00-1.00]

C

	cg16767801			cg23642047			cg16767801 + cg23642047	
Proportion	Sensitivity	Specificity	Proportion	Sensitivity	Specificity	Proportion	Sensitivity	Specificity
10%	1.00 [1.00-1.00]	0.14 [0.14-0.14]	10%	0.96 [0.94-0.98]	0.12 [0.11-0.13]	10%	1.00 [0.98-1.00]	0.14 [0.13-0.14]
20%	0.97 [0.94-0.98]	0.27 [0.26-0.28]	20%	0.92 [0.91-0.93]	0.25 [0.25-0.25]	20%	0.99 [0.96-1.00]	0.28 [0.27-0.29]
30%	0.93 [0.91-0.94]	0.40 [0.39-0.41]	30%	0.90 [0.89-0.91]	0.39 [0.39-0.39]	30%	0.97 [0.96-0.98]	0.42 [0.42-0.43]
40%	0.91 [0.91-0.91]	0.54 [0.54-0.54]	40%	0.85 [0.81-0.87]	0.51 [0.50-0.53]	40%	0.96 [0.96-0.96]	0.57 [0.57-0.57]
50%	0.90 [0.89-0.91]	0.69 [0.68-0.69]	50%	0.76 [0.74-0.78]	0.62 [0.61-0.63]	50%	0.96 [0.96-0.96]	0.72 [0.72-0.72]
60%	0.85 [0.83-0.87]	0.82 [0.81-0.82]	60%	0.66 [0.65-0.67]	0.73 [0.72-0.73]	60%	0.96 [0.96-0.96]	0.87 [0.87-0.87]
70%	0.81 [0.81-0.81]	0.95 [0.95-0.95]	70%	0.57 [0.56-0.57]	0.83 [0.82-0.83]	70%	0.88 [0.87-0.89]	0.98 [0.97-0.98]
80%	0.61 [0.59-0.61]	1.00 [0.99-1.00]	80%	0.46 [0.44-0.48]	0.93 [0.92-0.94]	80%	0.61 [0.61-0.61]	1.00 [1.00-1.00]
90%	0.30 [0.30-0.30]	1.00 [1.00-1.00]	90%	0.30 [0.30-0.30]	1.00 [1.00-1.00]	90%	0.30 [0.30-0.30]	1.00 [1.00-1.00]

Supplementary table 3: Replication of commercial or research methylation assays or previously published genome-wide studies

Gene	Test	Replication of a CpG at P value 0.05 x 10^{-8}
PAX1 DNA detection kit	iSTAT Biomedical, Taiwan	Yes
ZNF582	iSTAT Biomedical, Taiwan	Yes
FAM19A4	QIAsure Methylation Test (Qiagen, Germany)	No
miR124-2	QIAsure Methylation Test (Qiagen, Germany)	No
ASTN1	GynTect (Oncgnostics Jena, Germany)	No
DLX1	GynTect (Oncgnostics Jena, Germany)	No
ITGA4	GynTect (Oncgnostics Jena, Germany)	No
RXFP3	GynTect (Oncgnostics Jena, Germany)	No
SOX17	GynTect (Oncgnostics Jena, Germany)	No
ZNF671	GynTect (Oncgnostics Jena, Germany)	No
POU4F3	CONFIDENCE Marker (Neumann Diagnostics, Hungary)	No
EPB41L3	S5 CareLYFE (China)	Yes
ATP10A	El Zein et al, IJC, 2020	Yes
HAS1	El Zein et al, IJC, 2020	No
FMN2	El Zein et al, IJC, 2020	Yes
CA10	El Zein et al, IJC, 2020	Yes
DPP10-AS1	El Zein et al, IJC, 2020	Yes

RALYL	El Zein et al, IJC, 2020	Yes
SOX1	El Zein et al, IJC, 2020	Yes
NTM	El Zein et al, IJC, 2020	Yes
KCNIP4	El Zein et al, IJC, 2020	Yes
USP29	El Zein et al, IJC, 2020	No
CLVS2	El Zein et al, IJC, 2020	Yes
SOX11	El Zein et al, IJC, 2020	Yes
LINC01785	El Zein et al, IJC, 2020	No
MDGA2	El Zein et al, IJC, 2020	Yes
ASCL1	Verhoef, IJC, 2022	Yes
LHX8	Verhoef, IJC, 2022	Yes
CADM1	Hesselink 2011/Verhoef 2015	
MAL	Hesselink 2011/Verhoef 2015	

Supplementary Figures

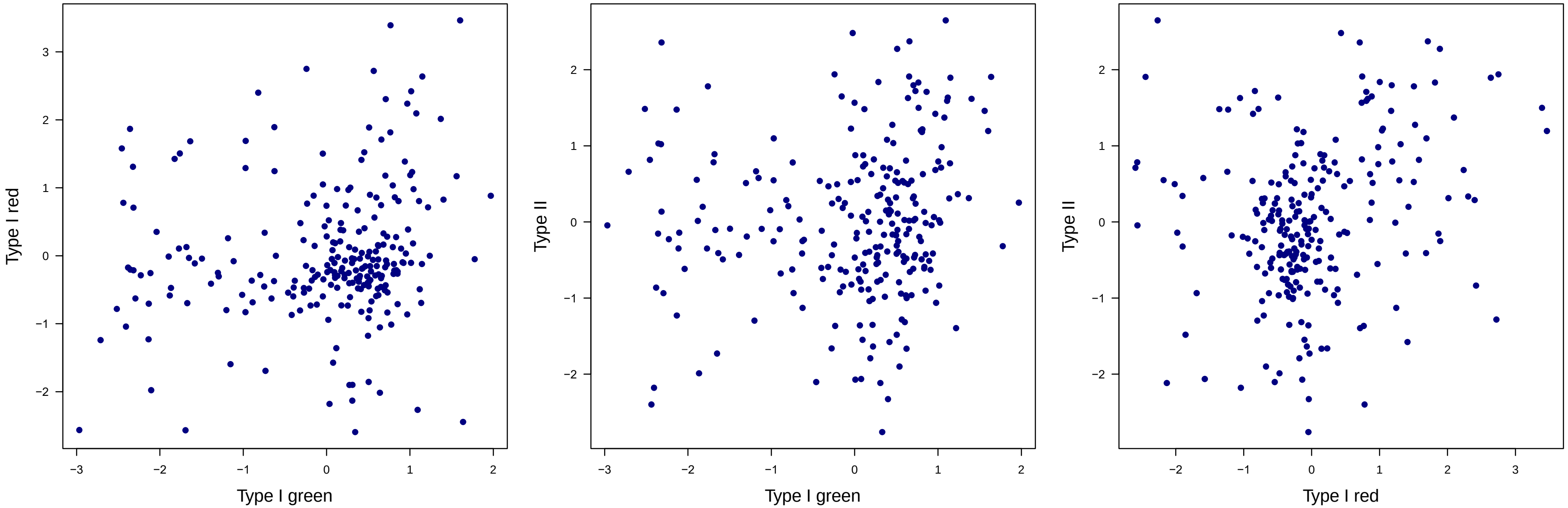

Supplementary Figure 1. Visualisation of technical metrics for Illumina 850K probes. Samples are represented along three variables measuring the average signals along the three types of beads (type I green, type I red and type II). No outlying observations were observed.

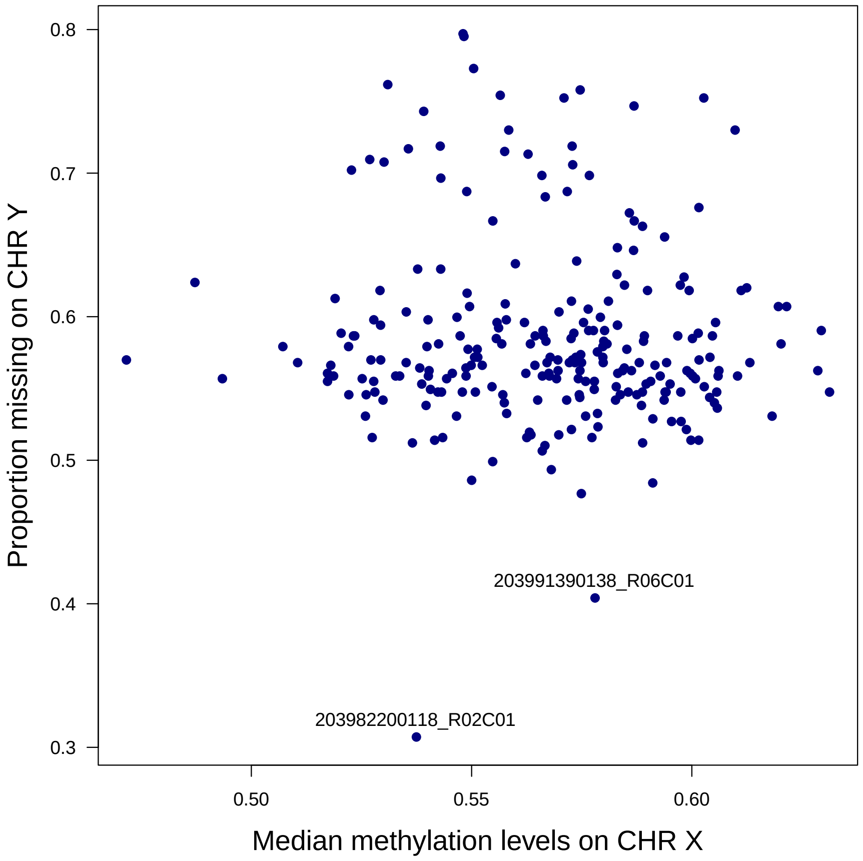

Supplementary Figure 2. Visualisation of the proportion of missing values on chromosome Y as a function of median methylation proportions on chromosome X. Women are expected to show higher proportions of missing values on chromosome Y and higher median proportions on chromosome X compared to Men.

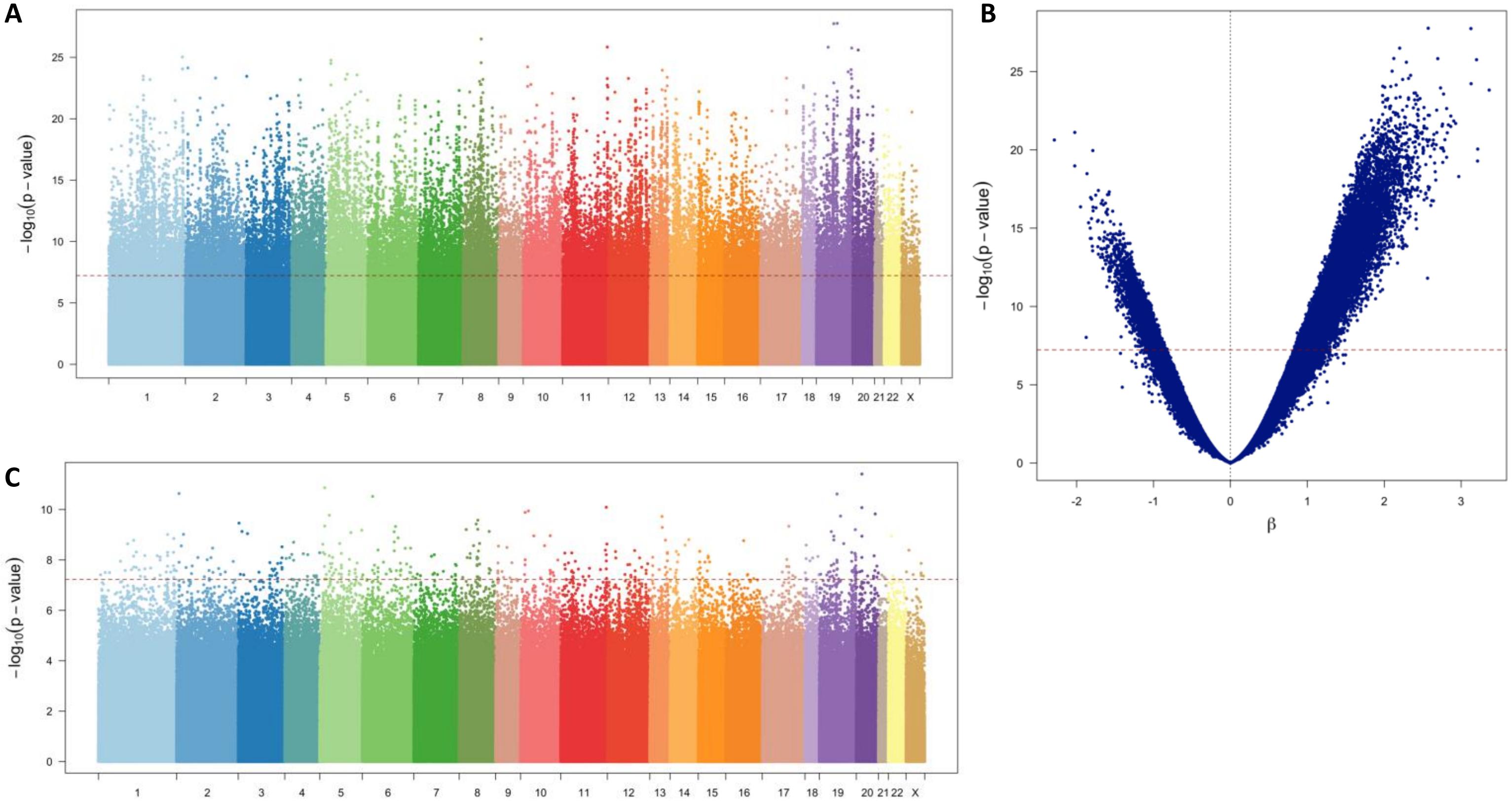

Supplementary Figure 3: Association between CpG sites and CIN3 (N=73) or ICC (N=54) status (N=114 controls) unadjusted versus adjusted for HPV status in the study population. (A)Manhattan plot showing the P-values, measuring the strength of association, derived from logistic models **unadjusted for HPV status** and represented on the -log10 scale (Y-axis). CpG sites (N=843,611) are ordered by their position on the genome (X-axis). (B) Volcano plot demonstrating distribution of methylation beta-values by -log10(P-value) unadjusted for HPV status where 0 represents the null value and the red dashed line represents the Bonferroni-corrected EWAS significance level. Genome-wide a greater gain in methylation is observed in association with CIN3 or ICC case status across the assayed CpG sites on the EPIC array. (C) Manhattan plot showing the P-values, measuring the strength of association, derived from logistic models **adjusted for HPV status** and represented on the -log10 scale (Y-axis). CpG sites (N=843,611) are ordered by their position on the genome (X-axis). The epigenome-wide significance level is set to the Bonferroni-corrected threshold (horizontal red line).

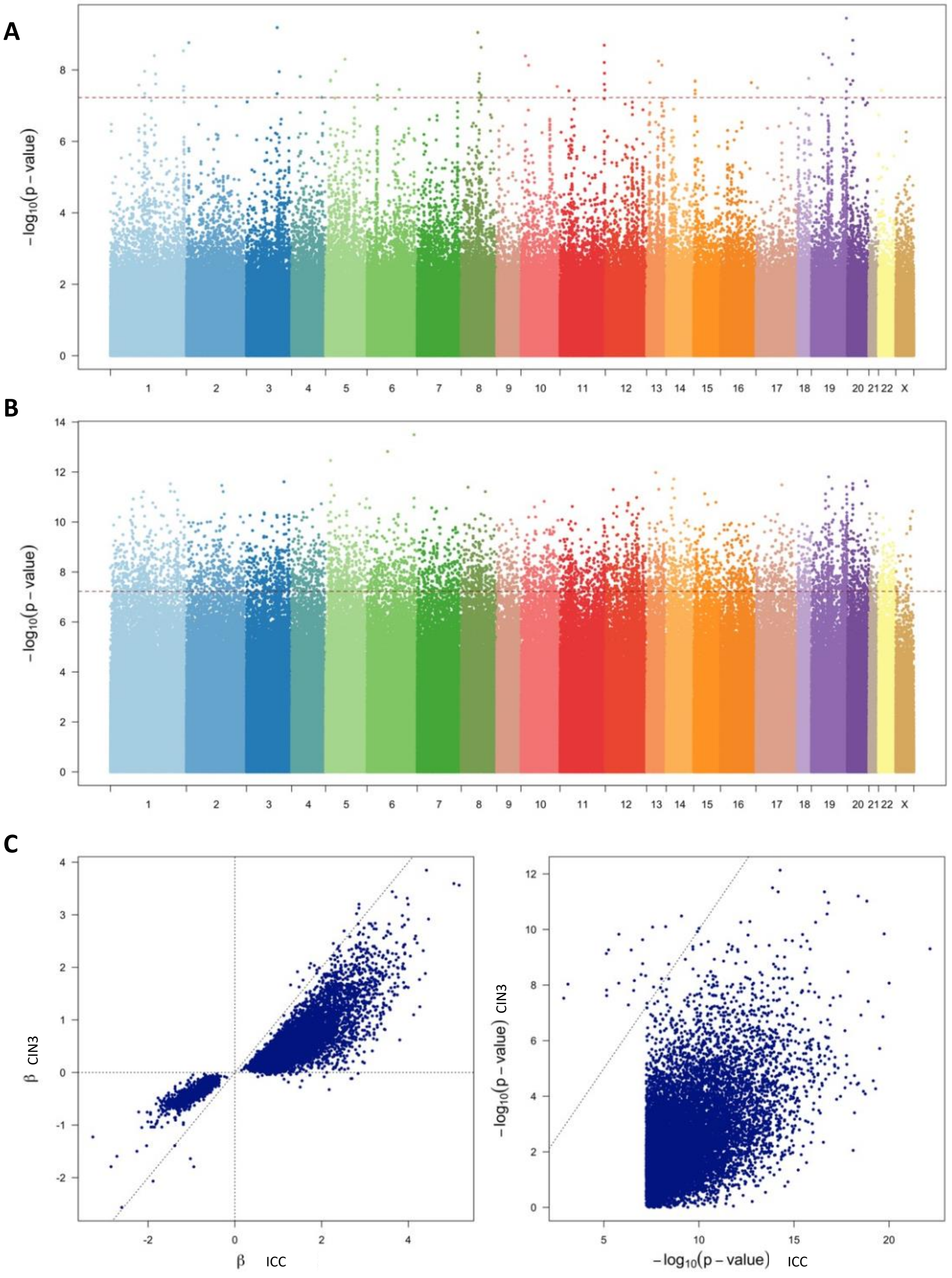

Supplementary Figure 4. Manhattan plot showing the P-values, measuring the strength of association, derived from logistic models adjusted for HPV status and represented on the -log10 scale (Y-axis) for CIN3 only (A) and ICC only (B). CpG sites (*N*=843,611) are ordered by their position on the genome (X-axis). The epigenome-wide significance level is set to the Bonferroni-corrected threshold (horizontal red line). Scatter plots of beta values (C, left) and -log10(P-value) (C, right) for all CpG sites identifies as statistically significant in the main analysis (N=409), are plotted separately for CIN3 (y-axis) and ICC (X-axis)

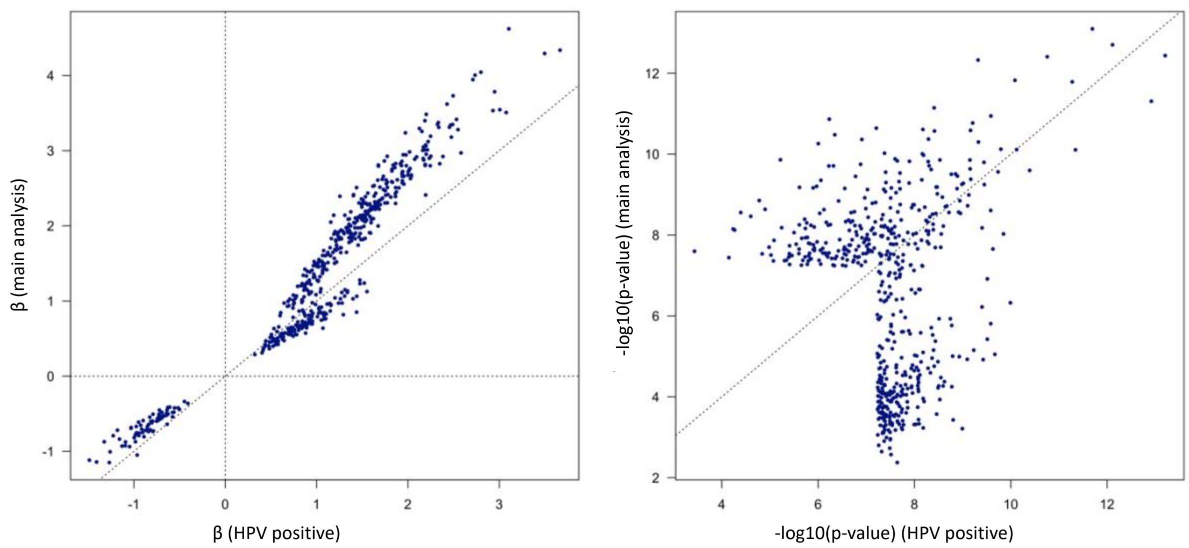

Supplementary Figure 5: Scatter plots showing the correlation of beta methylation values (left) and -log10(P-value) (right) for Bonferroni significant CpG sites (N=409 CpG) from logistic models of the main analysis with the CIN3/ICC status as the outcome adjusted for HPV status (X-axis, N=114 controls and N=127 cases) or restricted to HPV positive participants (Y-axis, N=25 controls and N=111 cases)
